# Ultradeep analysis of tumor heterogeneity in regions of somatic hypermutation

**DOI:** 10.1186/s13073-015-0147-1

**Published:** 2015-03-12

**Authors:** Janice M Spence, John P Spence, Andrew Abumoussa, W Richard Burack

**Affiliations:** Department of Pathology and Laboratory Medicine, University of Rochester Medical Center, Rochester, NY 14642 USA; NGS Tech, 936 Little Pond Way, Webster, NY 14580 USA; Department of Computer Science, University of Rochester, Rochester, NY 14642 USA

## Abstract

Tumor heterogeneity is of growing importance in the treatment of cancers. Mutational hot spots are prime locations for determining number and proportions of low variant allele frequency (VAF) tumor subclones by next generation sequencing. Low VAF detection is complicated by poor mapping efficiency in regions with high mutation density. Our Deep-Drilling with iterative Mapping (DDiMAP) method retains variant allele patterns to aid in single nucleotide variation detection and generation of additional reference alleles, with remapping increasing coverage of highly mutated regions to capture data critical to heterogeneity analysis and enhancing sensitivity. DDiMAP outputs variant patterns with frequencies, enabling rapid phylogenetic analysis of ongoing mutation.

**Electronic supplementary material:**

The online version of this article (doi:10.1186/s13073-015-0147-1) contains supplementary material, which is available to authorized users.

## Background

Next generation sequencing (NGS) techniques are widely used to explore diverse areas in the study of cancer, including identification of driver mutations, measurement of tumor heterogeneity, investigation of genetic susceptibility, and characterization of mutational motifs to better understand underlying mutational processes. Though cancer has long been considered a monoclonal process, recent studies show that ongoing mutagenesis generates subclonal populations whose numbers wax and wane depending on the variant’s relative evolutionary fitness [1-5]. Tumor subpopulations possessing driver mutations conferring a selective advantage are the proposed source of tumor progression and acquired chemo-resistance [4,6-11]. In addition to rare driver mutations of obvious importance, there are numerous passenger mutations found at low allelic frequency within the tumor population, presumably due to ongoing genetic stress within the tumor that results in tumor heterogeneity [5,7,12,13]. Several studies have suggested that the level of tumor heterogeneity itself may serve as a prognostic indicator [14-16]. Thus, sequencing and analysis methods designed to identify and characterize tumor diversity and evidence of ongoing mutation may provide a relative measure of the mutagenic stress and/or inadequacy of the DNA repair systems within a given tumor with the potential to inform clinical care.

Follicular lymphoma (FL), a B-cell lymphocytic cancer, is particularly well-suited for development of an approach to measure tumor heterogeneity. First, it provides a positive control for genetic heterogeneity in the form of the uniquely rearranged *IGH* loci which encodes for immunoglobulins, a tumor-specific marker known to be subjected to ongoing somatic hypermutation (SHM) [17-20]. Second, the activation induced cytidine deaminase (AID)-mediated mutagenic process responsible for SHM is well characterized with regard to sequence motif and substrate specificity [21-23], providing a mechanism to evaluate the validity of SNV calls, especially those at low frequencies. Third, there are reported genes outside the *IGH* loci that may be subjected to AID-mediated aberrant somatic hypermutation (aSHM) in B-cell lymphomas [24-30], providing selected regions with a high likelihood of significant mutational events for our targeted re-sequencing approach.

**Figure 1:**
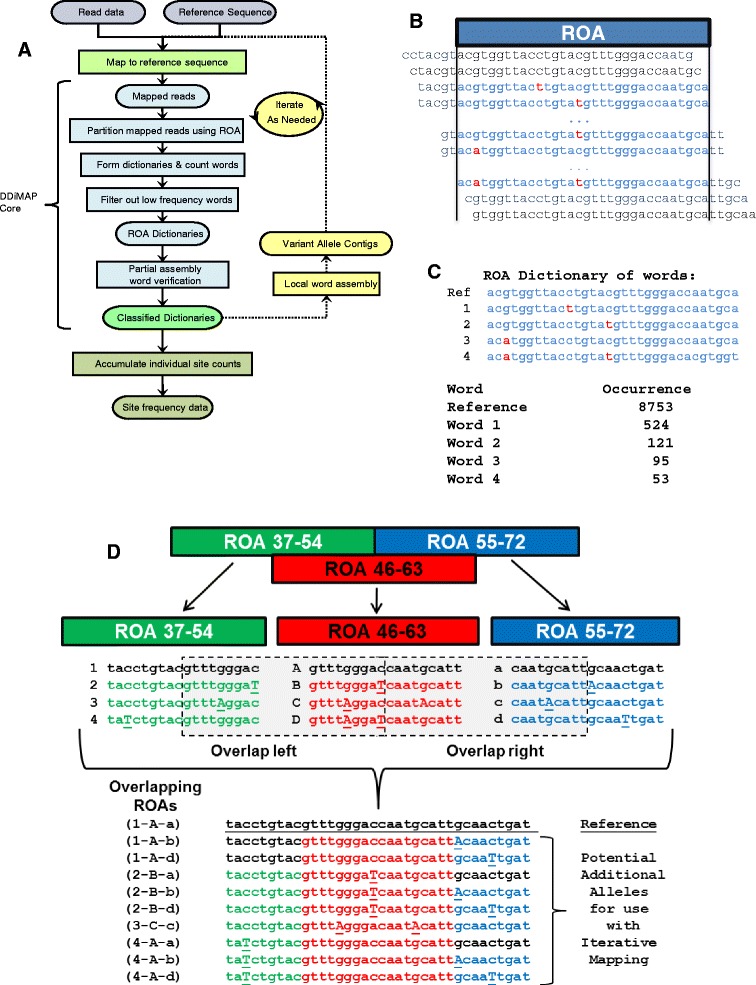
**Deep-Drilling iterative Mapping (DDiMAP) flowchart. (A)** This overview schematic illustrates the novel components in a DDiMAP pipeline. Key points include partitioning of reference sequence into computational units called regions of analysis (ROAs), with mapped reads uniquely assigned to ROAs using alignment information within bam files. Variant sequence patterns are collected in each ROA, forming a ‘dictionary’ of unique ‘words’ which are retained based on frequency thresholds. Retained words are partially assembled with words from overlapping ROAs in a cross-verification process. Partially assembled sequences containing variant sequence patterns may be used as additional reference sequences for the optional remapping of reads, a process that is repeated until no new variants above a coverage dependent threshold are observed. For variant identification, data from all ROAs are tallied at each location from the verified dictionary-based sequences. **(B)** Reference sequence is partitioned into abutting ROAs. Reads are assigned to an ROA based on their ability to completely cover the ROA, maintaining the contained read segments (blue letters) while discarding excess (gray letters). **(C)** ROA analysis includes counting all read segments matching observed word sequence patterns. This compresses all read data from each ROA into a listing of unique words with count of occurrences in each read direction. **(D)** An ROA collection is a pair of tracks of abutting ROAs that overlap by half, with reads assigned to one and only one track. Overlapping dictionaries facilitate partial assembly of sequences to form longer sequence fragments containing observed variation from the initial reference sequence that are added to enhance mapping of reads that contain a high density of variation. Additionally, comparison of overlapping dictionary entries formed using independent sets of overlapping variant sequences provides an independent cross-validation of variant sequences for SNV identification. See text and Additional file 1 for complete details.

The most productive regions to look for signs of ongoing mutagenesis are mutagenic hot spots. Close linkage to tumor specific mutation patterns is necessary to unambiguously identify low frequency passenger mutations as evidence of ongoing mutation within shifting dominant tumor subclones. The specific challenge here is accurate identification and quantification of mutations with low variant allele frequency (VAF <1%) in genomic regions with high density of variation from reference [31]. We found this is a two-part problem: the well described issue of distinguishing true single nucleotide variations (SNVs) at low frequencies that represent ongoing mutagenesis from process errors and the less well publicized problem of accurately mapping reads from highly divergent genomic regions representative of aSHM/kataegis, compounding the problem of identifying additional low frequency events in these regions. Our solution, which we call Deep Drilling with iterative Mapping (DDiMAP), is a multi-pronged approach that includes the use of sufficient numbers of tumor cells to adequately sample rare events, ultra-deep sequencing (>10,000×) of regions of aSHM/kataegis, and maintaining subclonal specific sequences throughout the entire process for multiple uses. The core of DDiMAP takes mapped reads and analyzes them in groups (regions of analysis (ROA)) to detect patterns in the read data (‘words’) arising from allelic variants in the presence of instrumental noise. It maintains these word patterns to assist in both iterative remapping and low frequency variant calling (Figure 1). Other programs, such as SRMA [32], IMR [33], and iCORN [34], use data-driven alternate reference sequences followed by remapping to identify a consensus genomic sequence. In contrast, DDiMAP specifically maintains ROA-based collections of these diverse sequence patterns in ‘dictionaries’ to identify and quantify subclones within a tumor population, polyploidal organisms, or other mixed populations. We developed this approach with empirical data from a PCR-based targeted re-sequencing study of follicular lymphoma (FL) using SOLiDv4, and also applied it to a PCR-based *IGH* sequencing study from Hodgkin lymphoma (HL) using Illumina MiSeq data. We evaluated its technical performance using synthetic combinations of empirical data as well as simulation data of ongoing mutation in a genetic region with high density of mutation incorporating simulated Illumina HiSeq process errors.

## Methods

**Figure 2:**
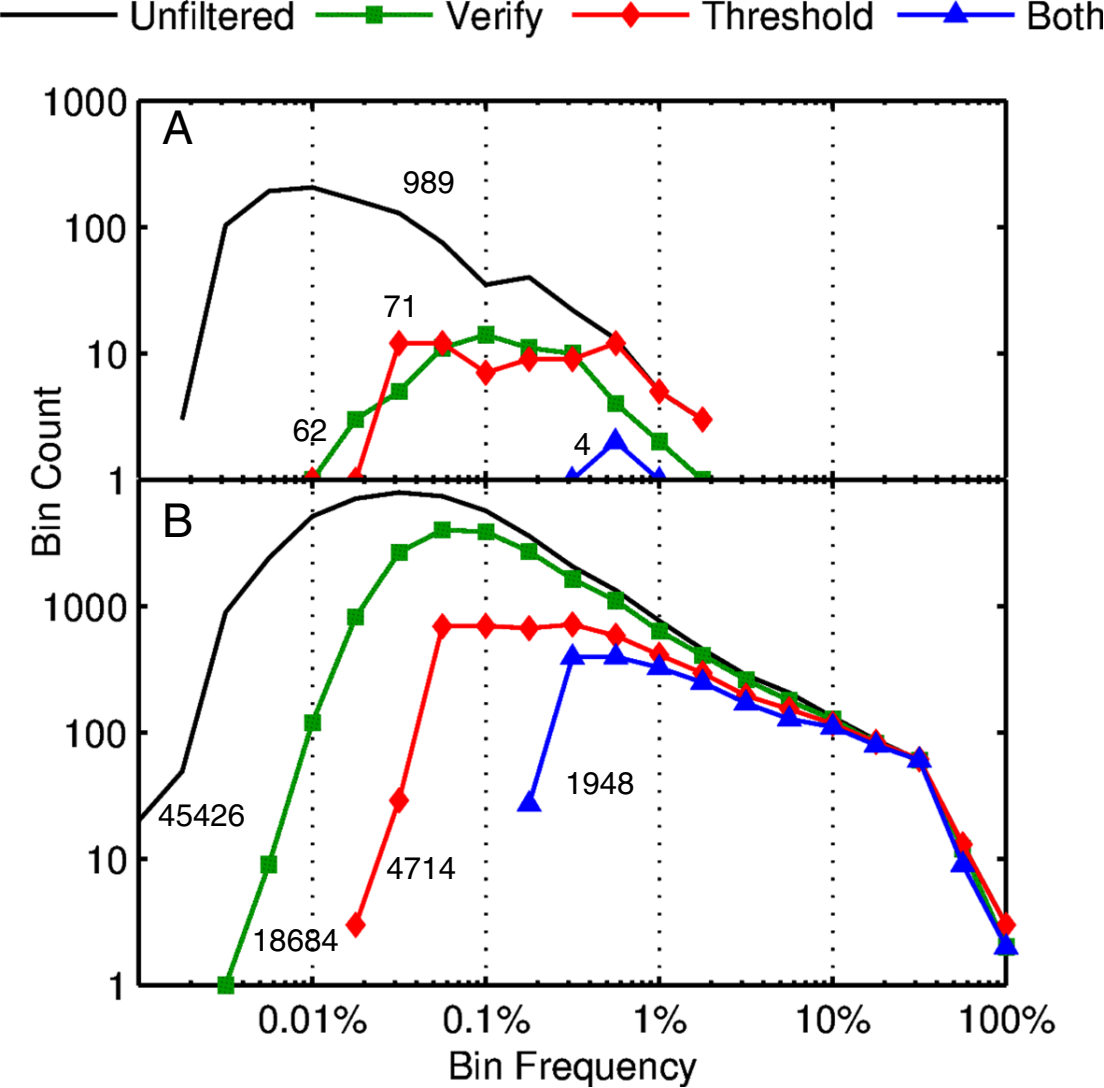
**Threshold and cross-validation filters effectively remove signal noise while retaining true variant calls. (A)** Negative control plasmid fragments (pBluescsript II KS) were spiked into FL specimen pooled amplicons at 1/10 the concentration of the individual targeted gene amplicons. Aggregating the aligned read data from the 12 FL specimens after mapping with BFAST, resulted in an average location coverage of 45,000×, in which there were 989 observed raw candidate SNV calls discovered (black line). Application of either the bidirectional minimum word threshold frequency filter at 750 ppm (red diamonds) or by cross-verification (green squares) alone dropped the candidate SNV call counts to less than 10% of the raw calls, while application of both filters had a synergistic effect, eliminating >99.5% of the initial calls (4/989 - blue triangles). **(B)** The *IGH* data from the 10 FL specimens was aggregated in a similar manner, resulting in 45,426 raw candidate SNV calls (black line) with a lesser reduction due to cross-verification (18,684 remaining or 41% - green squares), a similar reduction due to thresholding (4,714 or 10% - red diamonds) and a combined reduction to 1,948 final SNV calls (>4% - blue triangles) following application of both threshold and cross-verification filters. Note that the combination of filters retains the vast majority of SNV calls which were present at frequencies >1%. Reads were mapped to the Sanger-level sequence of the clonal *IGH* from each FL specimen and represents SHM generated variation around the clonal sequence.

**Figure 3:**
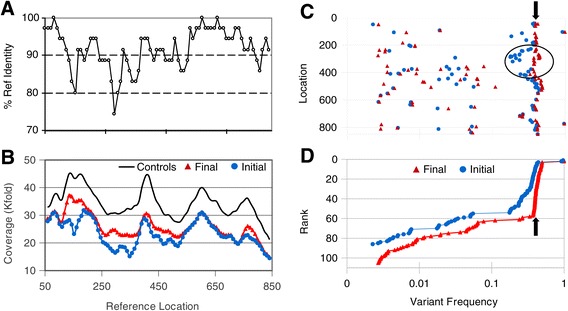
**Iterative mapping increases accuracy of total SNV calls and frequency estimations from FL-128** ***BCL2*** **region. (A)** Percent identity (35 base moving average) of *BCL2* from FL-128 (Sanger sequence) to its GRCh37.p10 reference has a minimum value of 75% identity. **(B)** Local coverage relative to reference location for initial BFAST mapping (blue circle) and converged iterated BFAST mapping (red triangle) shows improved coverage in areas with lower percent identity; coverage in controls (black line), offset by 5,000, shows typical non-uniform coverage pattern. **(C)** Frequency of variants (# mutated reads/total # reads) called by analysis of initial BFAST (blue circle) and final iterated BFAST (red triangle) are plotted versus reference location using frequency along the horizontal axis to facilitate comparison to **(D)**. These alignment data show a wide range of frequencies and a general increase in detected frequency with iterative mapping, most notable in regions where iteration increased coverage (circle). Iterative remapping also identified an additional 19 SNVs (22% increase). Note that a large number of mutations share a common frequency, representing the founder genotype of the current most frequent clone (MFC) in this population (black arrow). **(D)** Cumulative frequency distribution data plotted as rank (1 to 86 for initial BFAST, 1 to 105 for converged iterated BFAST, high to low frequency) versus variant frequency show the increase in the number of variants detected and a tighter distribution of MFC genotype frequencies upon iteration (black arrow). Two homozygous SNPs are present in this sample.

DDiMAP is designed to search through relatively small regions of the genome at a very high level of sequencing coverage to identify and quantify genetic subpopulations. The software developed for the analysis step of this process (Figure 1A) can accept output from any aligner that generates bam files, even allowing use of different aligners for each iterative run. Primary components of DDiMAP analysis include: (1) dividing the reference sequence into computational units (ROAs) and uniquely assigning mapped reads to these ROAs (Figure 1B); (2) for each ROA, generating the collection (dictionary) of unique read sequences (words) along with associated frequency statistics for both threshold and cross-verification filtering to remove false variant calls (Figure 1C); (3) using partial assembly of high confidence words to generate additional alternate reference sequence fragments for iterative remapping, typically repeating the process until no novel high confidence variants are found (Figure 1D); and (4) compiling dictionaries containing verified words for identification of variants with associated frequencies at individual reference locations. In this way, thousands of reads that are assigned to any given ROA are reduced to a dictionary of words and their associated strand tallies.

### Generating computational units: region of analysis (ROA)

ROAs are obtained by partitioning a reference sequence into two tracks of overlapping segments, generated by choosing an analysis start location on the reference sequence and selecting an ROA length, with the ROA overlap defined as one-half the ROA length. We define a ‘collection of ROAs’ as two tracks of abutting segments of the reference sequence with the starts of the two tracks offset by the overlap length (Figure 1D). Mapped reads are uniquely assigned to an ROA based on their start position and CIGAR string information to determine which bases in the reference sequence, and thereby which ROA(s), are covered by the read (Figure 1B). If more than one ROA is covered, we assign the read to the ROA closest to the start of the read according to its read direction. The read is trimmed to fit the ROA unless the read is sufficiently long to span adjacent, non-overlapping ROAs, then the read is split among them and trimmed. Currently, reads containing indels are handled by eliminating inserted sections that do not correspond to bases in the reference sequence and by inserting a missing data symbol (-) in deleted segments for each base deleted.

### Multistep filtering process

We designed a multistep procedure to selectively retain words that are more likely to contain true sequence variations. This procedure includes a threshold filter to eliminate random procedural errors followed by partial sequence assembly to cross-validate observed patterns of variation in words.

#### Threshold filtering

Threshold filtering only retains word sequence patterns that are observed on both strands above a minimum count threshold; eliminating most words containing false variants arising from random instrumental or low frequency PCR-based errors (strand bias). The threshold is coverage dependent with an absolute minimum count used in regions with relatively low coverage and a relative minimum count based on a proportion of the coverage used in regions with higher coverage. This threshold can be set at a higher level to provide more stringent filtering for generation of alternate reference fragments or at a lower level for use in final variant candidate identification.

#### Sequence verification through partial assembly

The second step utilizes partial assembly of words from dictionaries from overlapping ROAs to validate sequence patterns as a cross-verification process (Figure 1). This step leverages the unique assignment of a read to a single ROA track which guarantees that sequence data from two overlapping ROAs within the same collection come from independent sets of reads. To perform sequence cross-verification, each unique word in an ROA dictionary is split, and the half words are compared to the corresponding half words from their overlapping ROAs (Figure 1D). Words that have matching half sequences from both overlapping ROAs are ‘fully verified’, words that only match on one side are ‘partially verified’, while words that have no match with either overlapping ROA are ‘unverified’. The outcome of this process is the generation of read segments with categorized levels of confidence in sequence patterns that will be used both in the iterative remapping process and to identify final candidate SNVs after completing the last round of remapping.

### Iterative remapping

Alternate reference fragments are constructed from sequence and assembly information generated during the partial assembly step for cross-verification as shown in Figure 1D. For each ROA, words in its dictionary (red text) that are tagged as fully verified are extended in both directions by assembling all combinations of matching verifier words from overlapping ROA dictionaries (green and blue text), resulting in a fragment that is twice the ROA size. Alternate reference fragments may also be generated from words that are not fully verified. In our assembly process, words that are partially verified may be extended in their verified direction using matching verifier word(s) (green and blue text) and in the other direction by appending reference sequence (black text) and words that are non-verified may be extended in both directions using reference sequence (black text). Provisional acceptance of non-verified words for the purpose of providing alternate allele fragments permits extension into regions with low mapping coverage due to alignment failure. The assembly process may of course be implemented using alternate assemblers starting from the words in the dictionaries.

Since mapping algorithms are capable of aligning reads containing variation, it is not necessary to introduce all the variants that might be present in a sample to enhance mapping sensitivity. Typically we use a stringent iterative threshold filter setting for generating additional reference fragments to limit introduction of false discovery events. Alternate reference fragments are introduced into the enhanced reference sequence collection for the next mapping iteration by choosing all extended fragments built around fully verified words and optionally choosing extended fragments built around partially verified or unverified words only if the central word accounts for a high proportion of its ROA coverage.

This process of generating additional reference fragments and mapping using the enriched reference sequence collection is repeated until no new words are observed above the iterative threshold setting. Once iterative mapping has converged, it is necessary to collect all the mapped read data from the multiple reference fragments into a common ROA dictionary based on reference location, summing variant words and coverage data from all reference fragments. The final analysis is performed at a more permissive threshold setting in dictionary formation to allow discovery of rare variants, capitalizing on the enhanced mapping sensitivity provided by the enriched reference sequence collection. See Additional file 1 for an in-depth discussion for selection of ROA length and other adjustable parameters within DDiMAP.

### DDiMAP sample software

We have posted sample source code implementing the DDiMAP methodology online [35]. This code accepts an input bam file and the reference sequence file used for its creation and several command line parameters for ROA size and thresholds and produces several files as output. The dictionary file, which is csv formatted, contains the word patterns with strand counts and verification status for all ROAs and reference sequences. The identified variant csv file lists the variants with frequency and local coverage. The coverage csv file contains total coverage at each position represented in the dictionary. The allele fragment file is in fasta format with an identifier for each fragment indicating its reference sequence and location. Sample Python scripts and shell scripts that employ the DDiMAP sample code to perform iteration are also included that illustrate how to implement an iterative scheme using other mappers. See Additional file 2 for a complete DDiMAP user guide.

Methods and Materials associated with generation and processing of PCR amplicons for targeted re-sequencing is presented in Additional file 1.

The data from this study have been uploaded to the Sequence Read Archive (NCBI) and are available for download under accession SRP055160. The specific reference sequences for these data can be found in Additional file 3.

## Results and discussion

DDiMAP increases the sensitivity of SNV calls while maintaining precision through two synergistic processes of filtering and remapping. The filtering phase uses sequence frequency thresholding and a cross-verification procedure to eliminate signal noise inherent in massively parallel sequencing. The iterative remapping stage uses successively identified high confidence variant sequences as additional reference allele fragments to enhance alignment of reads to highly mutated regions of the genome. Key to both processes is maintaining variant data information within word sequence patterns.

### Effect of filters

The primary filters include a threshold for minimum observed frequencies for each word in both sequencing directions and a cross-verification of the sequence through partial assembly of words from independent sets of reads. The empirical source of the thresholds and their efficacy when combined with cross-verification is evident from data using a plasmid fragment with no expected variation as a negative control and *IGH* sequence from FL specimens containing a high number of read variants representing ongoing SHM. The two primary filters removed the large number of low frequency variants typically associated with process ‘noise’ while retaining the higher frequency variants associated with true mutation patterns (Figure 2). Each filter process alone removed >90% from the plasmid negative control (pBluescript II KS fragment) and combined removed >99.5% of variant patterns while retaining 92% of *IGH* variants at frequencies >10%, 61% between 1.0 and 10%, and 15% between 0.15 and 1%, with nothing reported below 0.15% frequency.

Multiple observations support the validity of variants identified by DDiMAP within the FL/SOLiD dataset. First, Sanger sequencing confirmed >92% of variants that were identified and quantified by DDiMAP at frequencies >15%. Second, DDiMAP did not generate significant numbers of spurious variants, even at low frequencies, as evidenced by the complete lack of calls in some genetic regions while other regions from the same specimen show mutation rates >10% (see Additional file 4: Table AF1), demonstrating the ability of the filtering algorithms to remove low level process noise while retaining true signal. Third, analysis of the tumor specific variants in *BCL2* regions from FL specimens, at both high (≥15%) and low (≤1%) frequencies identified a consistent and highly significant bias towards the AID mutation patterns expected to be found in aSHM (see Additional file 4: Figure AF1: WRCY motif *P* <0.0001, WA/TW motif *P* <0.0012 by one-tailed Fisher’s exact test [36]), strongly indicating that the identified variants at both high and low frequencies are due to a common biological process and are not a computational artifact.

### Iterative remapping enhances read capture from regions with dense mutations

Read coverage irregularities are common in NGS, often due to issues associated with poor cluster formation from genetic regions with problematic sequence for polymerase amplification. In a heterogeneous population, lack of coverage due to poor mapping can overlap common coverage irregularities, obscuring the identification of subpopulations with significant regional coverage losses due to high density mutations. Figure 3B shows the irregular coverage pattern in *BCL2* from non-mutated controls (black line) compared to that observed for initial mapping of a highly mutated, highly heterogeneous tumor specimen FL-128 (blue circles), in which reduced local coverage correlates with mutational load (Figure 3A). Enriching the pool of reference sequences by including previously identified variant allele patterns, followed by remapping, improved mapping efficiency, raising coverage by 50% from regions with the greatest deviation (Figure 3B, red triangles), resulting in a coverage pattern indistinguishable from controls.

This enhanced mapping has significant effect on both total numbers of SNVs and SNV frequency estimations. The distribution of SNVs from FL-128, shown as SNV frequency versus reference location (Figure 3C), compares initial mapping (blue circles) with iterated to convergence results (red triangle) and demonstrates a scattering of low frequency SNVs between 0.3% and 10% with a clustering of SNVs at approximately 40% frequency, representing the genotype of the current most frequent clone (MFC) within this tumor (black arrow). A total of 19 new variants were identified following iteration (105 SNVs), a 22% increase over initial mapping (86 SNVs), with frequencies ranging from 0.17% to 40%, illustrating that even Sanger-level SNVs in densely mutated regions can be missed due to inadequate mapping. Overall, the SNV frequencies tend to increase with iteration, exemplified by the tightened distribution to the MFC founder genotype between reference locations 200 and 450 (black circle), corresponding to the region with the greatest increase of coverage (Figure 3B). The tightened distribution of the MFC frequencies is clearly illustrated in the cumulative frequency distribution plot (Figure 3D), suggesting that identification of subclonal populations by frequency comparisons may be both more accurate and precise following iteration.

To quantify the improvement in SNV frequency, we compared the SNP frequency estimates between initial and fully iterated mapped reads from the 12 FL specimens and 4 controls. The 54 homozygous SNPs showed frequency calls averaging 93.7% (range, 82.6% to 98.7%) on first mapping which increased to 98.8% (range, 93.5% to 99.7%) following iteration to convergence. Similar improvements were observed in 49 heterozygous SNP frequency calls averaging 47.0% (range, 30.8% to 54.7%) initially and rising to 50.8% (range, 44.4% to 56.4%) following iteration (both at *P* <0.0001, paired t-test [36]) (see Additional file 5). Thus there are two major advantages to iterative remapping: locally, enhanced detection of variants at both high and low frequencies in areas with dense mutation rates and globally, significantly better estimates of variant frequencies.

### Estimating functional capacity of DDiMAP - SOLiDv4 empirical data

**Figure 4:**
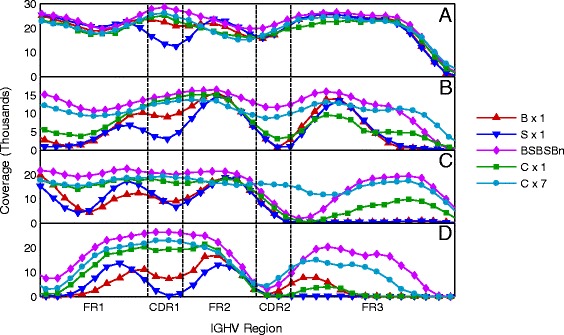
**Iteration greatly increases** ***IGHV*** **read coverage from empirical SOLiD data.** Coverage versus position within the FR1 to FR3 regions of the identified *IGHV* in four specimens with varying overall mutation rates. **(A)** Specimen 128, *IGHV1-18*, 7.3% mutation. **(B)** Specimen 134, *IGHV3-48*, 15.3% mutation. **(C)** Specimen 136, *IGHV3-15*, 17.3% mutation. **(D)** Specimen 132, *IGHV1-46*, 17.7% mutation. These plots show differences in read coverage between BFAST (B × 1), SHRiMP2 (S × 1), and CUSHAW3 (C × 1) mapping to germline sequences as the reference without iteration and the improvement in coverage obtained using iterative mapping wherein BFAST and SHRiMP2 are alternated twice, followed by additional BFAST iterations (BSBSBn) or CUSHAW3 for seven iterations (C × 7). Note how initial mapping coverage is lower in the CDR regions which typically are more highly mutated, but as the overall mutation rate increases, large regions including FR as well as CDR show severe loss of coverage due to the inability of the alignment programs to handle the clustered deviations from reference. See text for details.

To explore the capability of our word-based iterative approach over a wide range of high density mutation patterns, we used the natural experiment inherent in this FL/SOLiD dataset employing tumor *IGH* sequences. The rearranged and highly mutated *IGH* sequence is a unique biomarker for B cell tumors such as FL, and this sequence, with only minor variations, will be found in each cell derived from the tumor. To test the limitations of our DDiMAP process, we evaluated its ability to map reads derived from the tumor specific *IGHV* sequence to the appropriately matched non-mutated progenitor *IGHV* gene. Doing this allowed us to test DDiMAP’s ability to ‘uncover’ the observed Sanger level mutations. We evaluated the clonal *IGHV* segments between functionally defined regions of the *IGHV* genes: the highly conserved framework regions (FR1-3) which flank the hypermutated complementarity determining regions (CDR1-2) [37] for four FL specimens that express varying levels of mutational loads in their total *IGHV* sequence (10.3 to 17.9% variation). We evaluated three different color-space aligners, BFAST 0.7.0a [38,39], SHRiMP2.2.3 [40,41], and the recently published CUSHAW3 [42], each individually and also in combination, alternating BFAST with SHRiMP2 during the iteration process. As a baseline for comparison, we analyzed single round variant calls made from each mapping tool using the SNV caller within DDiMAP. We followed with iterative mapping using CUSHAW3 alone or with two sequential rounds of BFAST and SHRiMP2 followed by up to three rounds of iterative BFAST mapping (BSBSB_n_). Mapped coverage across the *IGHV* regions varied with the degree of deviation from the germline reference, and in all cases, significantly improved with iteration, independent of the aligner used (Figure 4), though some regions with marked variation remained difficult (Figure 4D).

However, in this test scenario, we are not identifying rare events which require high coverage but attempting to elucidate the predominant clonal *IGHV* sequence. Once a suitable level of mapping coverage is obtained, specific SNV identification is a better indicator of performance, especially with regard to the interpretative nature of color space NGS data. We used 25% as the minimum frequency to call any given SNV to allow identification of ambiguous bases observed in the Sanger sequence of the tumor *IGH*. The older color-space mappers we evaluated, BFAST and SHRiMP2, have mapping characteristics that led us to evaluate the utility of their sequential application. BFAST is designed to look for sequence variation, and allows a high number of mismatches so long as they do not lead to two or more consecutive base changes [38] while SHRiMP2 does permit the identification of pairs or triplets of consecutive base changes within its overall more conservative mapping allowance [40]. By alternating the mappers, the plan was to allow BFAST to capture enough isolated variants to enable SHRiMP2 to map reads to the alternate allele fragments, and that SHRiMP2 would add the adjacent base changes to the allele fragments, providing the necessary density of alterations for continued BFAST iterations. BSBSB_n_ was superior to both single pass BFAST and SHRiMP2 mapping and provided 100% recovery of *IGHV* sequence from specimen 128, with approximately 10% variation from germline sequence (see Additional file 4: Table AF2). In contrast, CUSHAW3 was able to recover the *IGHV* sequence from 128 without iteration. However, all mapping approaches failed to varying degrees as the sequences deviated by more than 15% from germline *IGHV*, with BSBSB_n_ identifying 85 SNVs while single round mapping from BFAST or SHRiMP2 identified 61 to 67 SNVs, respectively (see Additional file 4: Table AF2). As expected, the best predictor of BSBSB_n_ success in identifying a Sanger level base change was not the total deviation in sequence, but the pattern of mutation clusters. The lowest BSBSB_n_ recovery of Sanger level *IGHV* sequence was in specimen FL-136, identifying only 31% of the Sanger calls in a background of 17.3% *IGHV* variation, while it recovered 62% of Sanger calls in specimen FL-132 with an equivalent 17.7% *IGHV* variation; FL-136 has 26/48 Sanger identified mutations in adjacent bases (4 pairs and 6 triplets) while FL-132 has only 14/51 bases changes in adjacent bases (4 pairs and 2 triplets). Overall, CUSHAW3 had the best performance with high positive predictive value (PPV) in both the initial mapping and following iteration (>95%) while the false negative rate fell from 38% to 15% with iteration, recovering 85% (138/163) of the total SNVs identified by Sanger sequencing.

### Estimating functional capacity of DDiMAP - Illumina MiSeq empirical data

**Figure 5:**
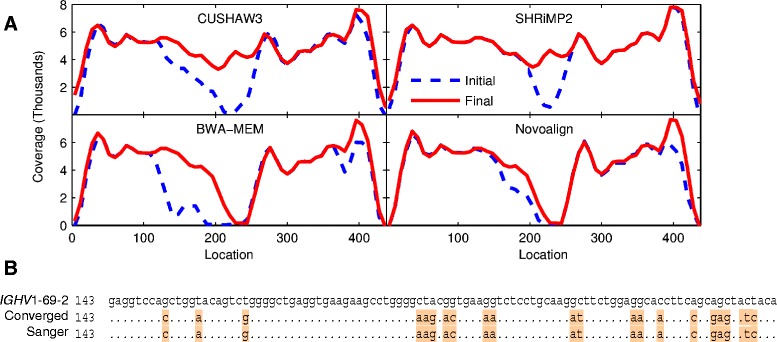
**MiSeq empirical** ***IGHV*** **read coverage improves with iteration and recapitulates Sanger sequence upon convergence. (A)** Coverage plots demonstrate influence of mapper selection and iteration for MiSeq data (50 base reads) of an *IGH* amplicon from a Hodgkin lymphoma specimen to its clonal *IGHV* region, *IGHV1-69-2*. The FR1-FR3 regions of this gene starts at location 143 and ends at 430. **(B)** Sequence alignment of the 100 nt region of highest divergence (21%) of HL-*IGHV* sequence from its corresponding germline sequence: *IGHV1-69-2*, the independently converged sequence from CUSHAW3 and SHRiMP2, along with the Sanger sequence from *IGH* amplicon from this specimen. Initial coverage of this region is low in part or all of this region for all four mappers shown and remains low after iteration in the highest density portion with the default settings for BWA-MEM and Novoalign.

A similar analysis, mapping *IGHV* read data to germline *IGHV* reference, was performed using Illumina MiSeq data from an HL specimen to determine if the observed initial mapping failures and restoration by iteration were platform specific behavior of the color-space mappers. We evaluated four nucleotide mappers (SHRiMP2, CUSHAW3, BWA-MEM [43,44], and Novoalign [45]) at default settings to map reads from a clonal *IGH* amplicon to its germline reference sequence (Figure 5A). All four mappers show a dramatic drop in initial coverage (dashed blue line) corresponding to the hypervariable CDR1 region of *IGHV* (location 218 to 241), with complete coverage restored following iteration to convergence (red line) with both SHRiMP2 and CUSHAW3, while BWA-MEM and Novoalign did not restore coverage in the region of highest divergence. Convergence occurred in seven iterations and the independently evolved sequences from both SHRiMP2 and CUSHAW3 completely recovered the Sanger sequence of this amplicon (Figure 5B), which had 21% local deviation (location 143 to 252) and 17.9% overall deviation (239/291 nt) to *IGHV4-61* [46]. This clearly shows that mapping inefficiencies are not platform specific but reflect the capability of the selected mapper to handle the degree of mismatch between reference and reads, and stresses the importance of proper mapper selection and optimization for the experimental goal. DDiMAP is a highly effective solution to low coverage in mutation hot-spots using mappers with a more balanced PPV/FPR while conservative mappers optimized for low false positive rate fail to map reads from these regions.

### Sensitivity of DDiMAP - Synthetic *IGHV* data

**Figure 6:**
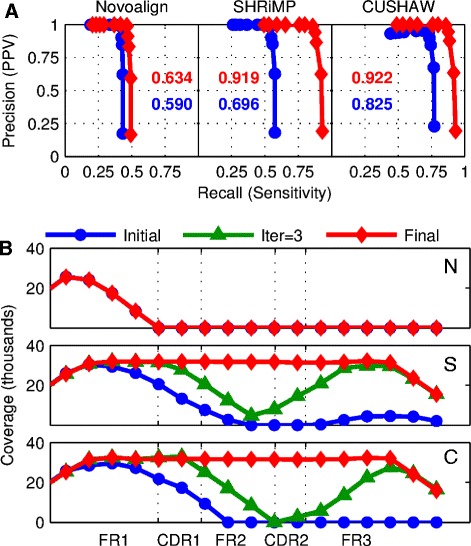
**Iteration improves sensitivity of DDiMAP variant identification and coverage of simulated Illumina HiSeq Data. (A)** Precision-Recall curves were generated by varying DDiMAP primary filter thresholds from 100 to 800 ppm and co-varying variant identification thresholds at 4× primary filter levels (see Additional file 1 for details). Performance is shown for initial and final iterations. A peak performance F1-score for each case is shown in matching color. Significant improvement in sensitivity is obtained with iteration for SHRiMP2 and CUSHAW3, with improvement in precision for CUSHAW3 as well. Novoalign, using default settings, had slightly improved sensitivity. DDiMAP attained its peak performance for all three mappers at a threshold of 300 ppm. **(B)** Coverage for SHRiMP2 (S) and CUSHAW3 (C) at the initial iteration, after the third iteration, and at the final iteration are shown. In these cases the final enhanced reference sequences contained the private founder clone sequence without creating false positive variants at any frequency. Coverage using Novoalign (N) did not improve with iteration.

To determine the sensitivity and precision of our DDiMAP approach, we used the Sanger-level *IGH* private sequence information from a highly mutated FL case as a biologically relevant mutation pattern in a simulation study incorporating both ongoing mutation from the initial sequence and simulated Illumina HiSeq error patterns in 100 base pair single-ended reads [47]. Ten generations of replication with an accompanying mutation rate of 1/10,000 bases resulted in 1,024 known allelic patterns containing variants at frequencies ranging from present in all patterns (100% frequency) to present in 1/1,024 (0.097% frequency). Reads were mapped to the *IGHV* germline sequence using SHRiMP2, CUSHAW3, and Novoalign and processed through DDiMAP to generate additional allele fragments for iterative remapping. Variants were identified with DDiMAP from both the initial and final converged mapping at a range of bi-directional acceptance thresholds (100 to 800 ppm) to generate precision-recall plots (Figure 6). Novoalign suffers from poor mapping efficiencies at both the initial and fully iterated runs, with low recall levels due to lack of coverage in these highly divergent regions. Both SHRiMP2 and CUSHAW3 show significantly improved sensitivity with iteration, resulting in essentially identical precision-recall curves that also match the curves obtained when the tumor-specific *IGH* Sanger sequence is used as the reference. In the FR1-FR3 region of *IGHV*, at the minimum threshold resulting in 100% PPV (400 ppm), the overall false negative rate is 8.0%, with 100% sensitivity (73/73) for mutations occurring at frequencies at or above 1/512 and 70.4% sensitivity (19/27) for mutations occurring at the minimal frequency of 1/1,024 (see Additional file 1 for experimental details).

### Sensitivity of DDiMAP - SOLiDv4 empirical data

**Figure 7:**
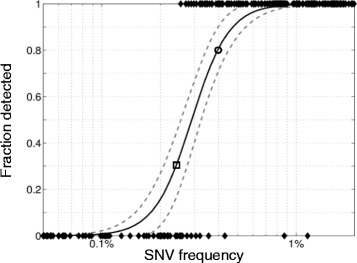
**DDiMAP shows high sensitivity, estimated with logistic model of mixed** ***BCL2*** **reads from SOLiD data.** Ten million reads were randomly selected from two specimens in proportions ranging from 1:31 to 31:1, mapped using BFAST and analyzed using the ROA threshold and verify procedure with a conservative threshold setting of 750 ppm applied in each direction (see Additional file 1 for experimental details). A set of 71 indicator mutations from the single specimens that had pure specimen frequencies ranging from 5% to 40% in *BCL2* were selected. The presence (1) or absence (0) of each of the indicator mutations in the various blends is plotted against their diluted frequencies on a log scale (non-informative data above 2% and below 0.05% are not shown). Also plotted is the logistic model (solid line) and 95% confidence limits (dashed lines). This model indicates that the method has 80% ±10% sensitivity for mutations occurring at a frequency of 0.4% indicated by the circle on the model plot. The data also indicate the method is unlikely to observe SNVs below the lowest observed indicator mutation frequency recovered from the blend (0.25%), where a modeled sensitivity of 30% ± 14% is marked by a square.

To assess the sensitivity of the word based analysis method based on experimental data, an *in silico* mixing experiment was performed by mixing SOLiD reads from two FL specimens at finely graded proportions ranging from 1:31 to 31:1 (see Additional file 1 for experimental details). The variants present in the pure specimen data were marked as present/absent at each dilution. A logistic model was fit using the logarithm of the product of the pure sample observed SNV frequency and the dilution factor as a predictor and the presence or absence of the variant as the dependent variable. The resulting model (Figure 7) indicates that the sensitivity of DDiMAP for the FL/SOLiD data set is 80% for SNVs occurring at a frequency of 0.4%, with a >99% probability of identifying SNVs occurring at 1.0%, obtained with a very conservative threshold setting of 750 ppm to limit false positive calls in FL specimens. These sensitivity estimates are consistent with those obtained with synthetic data and demonstrate that DDiMAP is capable of performing well at low VAF with empirical data.

### SNV identification by DDiMAP

We have already presented data demonstrating the effectiveness of the simple partial assembly approach to SNV calling used in DDiMAP; low false discovery rates in regions with high coverage levels (see Additional file 4: Table AF1), highly significant skewing of mutations to AID motifs at both high (≥15%) and low (≤1%) frequencies (see Additional file 4: Figure AF1) and high rate of validation for SNV calls ≥15% frequency by Sanger sequencing. However, we wanted to see how DDiMAP compared to a widely used, sensitive SNV caller that does not require matched normal samples. In studies of B-cell lymphomas, there are no matched normal genomic DNA for the uniquely rearranged *IGH* gene, and for tumor heterogeneity studies, the presence of private SNPs are not an issue as they simply add to the MFC genotype and are not misinterpreted as clinically significant ‘driver’ mutations. For these reasons, we selected VarScan2 [48,49] as our representative SNV caller for comparison purposes.

To evaluate the ability of DDiMAP to identify low frequency SNVs from an empirical data set, we performed an *in silico* blending experiment using reads from the 12 FL specimens mapped with BFAST (see Additional file 1 for experimental details). For each specimen, variants in *BCL2* identified by Sanger sequencing and/or VarScan2 analysis at a 1% threshold setting were used to establish ‘gold standard’ results. Each specimen has a readily identifiable founder genotype/most frequent clone (MFC) present at individual frequencies of 9% to 60%, generating a substantial number of variant allele frequencies (VAF) values from <1% to approximately 5% in the mixture. Additional non-founder level variants were present in the individual specimens at lower frequencies, leading to a total of 240 variants identified across the 12 FL specimens. Of these 240 ‘gold standard’ variants, there were 24 locations at which multiple variants were found across the 12 specimens, which required adjusting expectations within the mixed outcomes as VarScan2 at default settings only reports the most frequent variant at any given location. Of these 24 locations, 21 had two alternate base calls at a single location and three had three alternate bases at a single location, resulting in a maximum of 213 expected variants reported by VarScan2 from the mixture.

**Figure 8:**
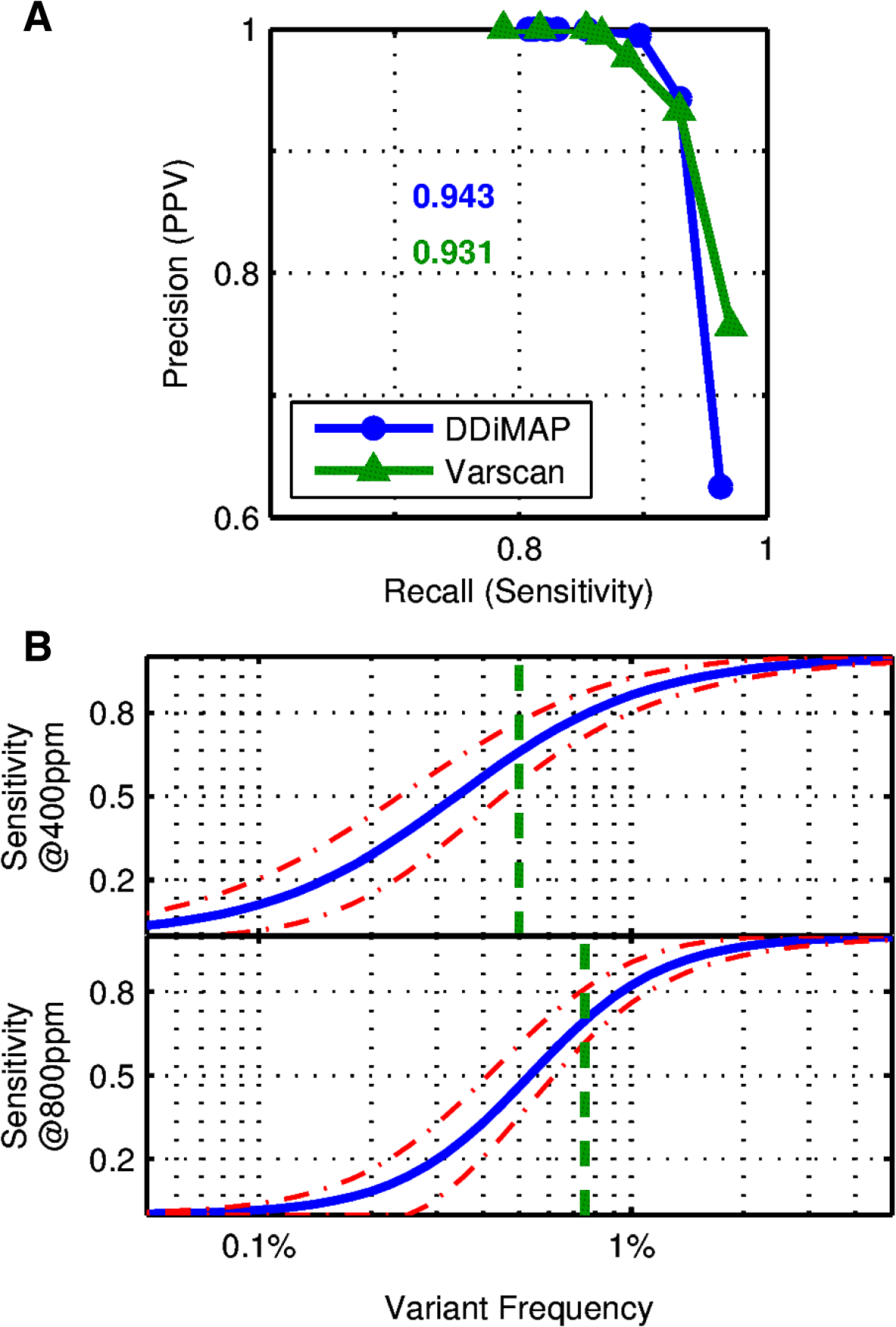
**Direct SNV candidate identification from DDiMAP filtered sequences compares favorably with VarScan2.** Mapped *BCL2* reads from the 12 FL specimens were individually analyzed by VarScan2 (1% threshold) and compared to Sanger sequencing of amplicons to identify founder clone genotypes for use as gold-standard SNV data (see Additional file 1 for details). Mapped SOLiD read data were pooled to generate a collection of variants at a wide range of low frequencies, with 24/240 at levels below 0.2% and 120/240 at frequencies below 2.1%. **(A)** Precision-recall curves were obtained using a threshold series in VarScan2 (0.1% to 1%) and DDiMAP (100 ppm to 800 ppm) to demonstrate their well-matched overall performance. VarScan2 requires a threshold of 0.5% to achieve 100% PPV with a corresponding sensitivity of 85.4% (F1-score = 0.92). Matching performance is obtained using DDiMAP with a threshold setting of 400 ppm. Peak F1-scores occur at lower threshold values of 0.2% for VarScan2 and 300 ppm for DDiMAP, reflecting different P-R trade-offs, with DDiMAP better for precision and VarScan2 better for recall and nearly matched to the 200 ppm position of DDiMAP. **(B)** DDiMAP logistic sensitivity models were obtained using two conservative thresholds that generate 100% PPV (400 ppm for upper plot, 800 ppm for lower plot) but different recall for the *BCL2* variants. At 400 ppm, 50% of variants at 0.33% and 80% of variants at 0.8% are detected while at the more conservative setting of 800 ppm, 50% of variants at approximately 0.5% and 80% of variants at approximately 0.9% are detected. VarScan2 thresholds of 0.5% and 0.75% provide matching overall performance, shown as vertical green dashed lines. Note how DDiMAP has increased sensitivity of low frequency variants with a reduced sensitivity at higher frequencies compared to theVarScan2 single hard threshold.

We analyzed this mixture with both VarScan2 and DDiMAP to identify variants at a range of thresholds (0.1% to 1% for VarScan2, 100 to 1,000 ppm for DDiMAP) to evaluate sensitivity and PPV. The resultant precision-recall curves (Figure 8A) show that the DDiMAP variant identification procedure that leverages the filtered words through partial assembly has highly similar overall performance outcomes to VarScan2. Based on their respective thresholds that yield 100% PPV, VarScan2 at 0.5% threshold has a false negative rate of 16.4% while DDiMAP at 400 ppm has a false negative rate of 15.5% when restricted to the calls reportable by VarScan2. Logistic models of the sensitivity dependence of DDiMAP on variant frequency at 400 and 800 ppm thresholds show that DDiMAP (blue) can identify low frequency variants below the VarScan2 hard threshold (green) with an acceptable trade-off between lower frequency true positive calls and higher frequency false negative calls (Figure 8B). It should be noted that the frequency distribution of detectable variants in this blended dataset is not reflective of an expected distribution for variants arising from ongoing mutation in FL, which would be dominated by lower frequency variants with a VAF <1% [31], dependent on the relative rates of mutation to growth. In such a case, DDiMAP would be expected to outperform VarScan2 because of its sensitivity below the VarScan2 threshold.

### Subclone identification

**Figure 9:**
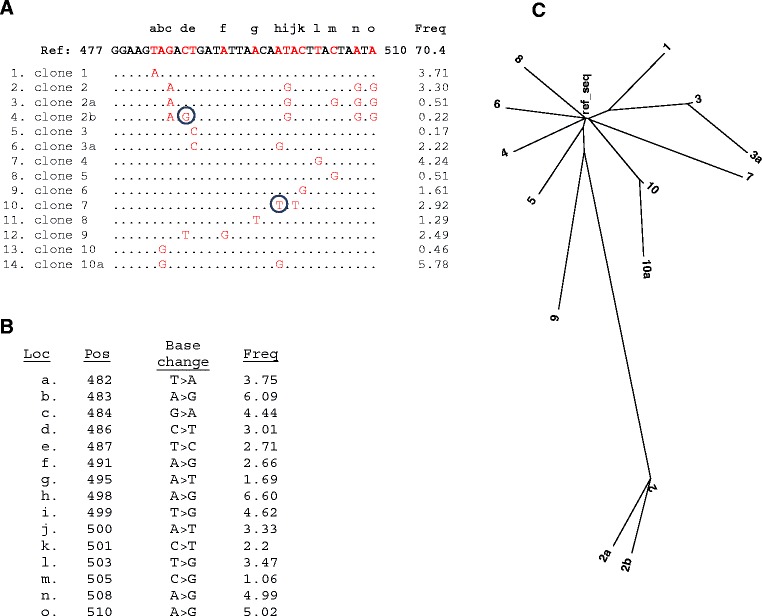
**Variant sequence patterns allow identification of 10/10 FL tumor populations from pooled** ***BCL2*** **reads. (A)** Reference sequence for *BCL2* ROA 447 to 510 and dictionary with associated frequencies (word occurrence/total coverage) are shown for the 12× blended *BCL2* analysis. Red letters in the reference sequence show 15 locations with identified base changes (a-o). The dictionary of verified words indicates reference identity with dots and changed bases with letters. Circled bases represent validated SNV calls not reported by VarScan2 due to presence of higher frequency variants occurring at identical locations. An inferred evolutionary interpretation of each word, based on phylogenetic analysis by maximum likelihood method (see 9C), precedes the word sequence, identifying 10 out of 10 known subpopulations within the mixture at this location, with three subpopulations showing additional mutations (clones 2, 3, and 10). **(B)** VarScan2 *BCL2* results at 0.5% threshold for locations covered by *BCL2* ROA 447 to 510, indicating reference location (a-o), associated base change and frequencies in the range of 1.06% to 6.60%. Note how ancestral or coincidental mutations are aggregated in the frequency determination, confounding the use of similar frequencies to identify subclonal specific mutation patterns. **(C)** This dendrogram was generated from DDiMAP dictionary: *BCL2* ROA 477 to 510 using MEGA6 [50], a freely-available online tool for evaluating evolutionary relationships based on sequence analysis. The evolutionary history was inferred by using the Maximum Likelihood method based on the Tamura-Nei model [51]. The tree with the highest log likelihood (-142.0600) is shown. Initial tree(s) for the heuristic search were obtained automatically by applying Neighbor-Join and BioNJ algorithms to a matrix of pairwise distances estimated using the Maximum Composite Likelihood (MCL) approach, and then selecting the topology with superior log likelihood value. The tree is drawn to scale, with branch lengths measured in the number of substitutions per site.

DDiMAP greatly simplifies subpopulation identification by providing a complete set of ROA dictionaries containing all verified words with associated frequency data (Figure 1C), provided in a dictionary.csv output file (see Additional file 2). Each ROA dictionary can be examined to identify the ROA with the largest number of verified words, representing the region of highest genetic diversity within a specimen. Using this approach on the 12-fold blended *BCL2* data, we found *BCL2* ROA 477 to 510 contained 14 verified words at VAF in the range of 0.17% to 5.78% (Figure 9A). While the 14 words imply the presence of 14 subclones, sequence comparison showed patterns of common mutations, suggestive of evolving clones. Molecular phylogenetic analysis by maximum likelihood method was performed on the DDiMAP dictionary output using MEGA6 [50], based on the Tamura-Nei model [51]. The tree with the highest log likelihood (-142.06), shown in Figure 9C, indicates 10 independent clones arising from the reference sequence. Sequences on lines 2 to 4 (Figure 9A), identified as clone 2, show a base sequence with four common mutations while lines 3 and 4 each have a single additional change; both are present at a lower frequency than the progenitor clone, consistent with ongoing divergent evolution of clone 2. Similar sequence relationships can be seen on lines 5 to 6 (clone 3) and 13 to 14 (clone 10) except here the ancestral clone proportion appears to be declining, consistent with the ‘clonal sweep’ pattern observed in other B-cell tumors [52]. Thus DDiMAP identifies 10 populations within this mixture, some showing ongoing mutation, which is the expected number based on gold standard SNV calls of the individual specimens in this ROA.

VarScan2 report for this same region of *BCL2* (Figure 9B) identifies most of the same base changes found by DDiMAP except for the two locations that have two different mutations at one site (Figure 9A, blue circled letters). Frequency-based determination of variant alleles is difficult due to the elimination of allele specific SNV correlation by mpileup [53] and similar analyses which consider SNV calls at each location independently. Additionally, evolutionary relationships are confounded by aggregation of frequencies from all identical SNV calls at a single location, as seen with the C > G mutation at location ‘k’, called by VarScan2 as 1.06% frequency but identified as part of two separate clones by DDiMAP (clones 2a and 6), each at 0.51%. These clones are not related, as the C > G is the sole mutation in clone 6 (line 9) but is the ongoing fifth mutation in clone 2 (line 3). This C at position ‘k’ is the targeted C of the AID motif, WRCY, and is thus more likely to be identically mutated in independent clones. Maintenance of SNVs in read sequence context is a powerful tool for VAF analysis, as the frequency of alleles is not obscured by aggregation of site-based frequencies, related alleles are cleanly delineated so their relationship can be readily observed, and determination of ongoing mutational activity on the current dominant clone can be clearly documented.

### Visualizing development of diversity

**Figure 10:**
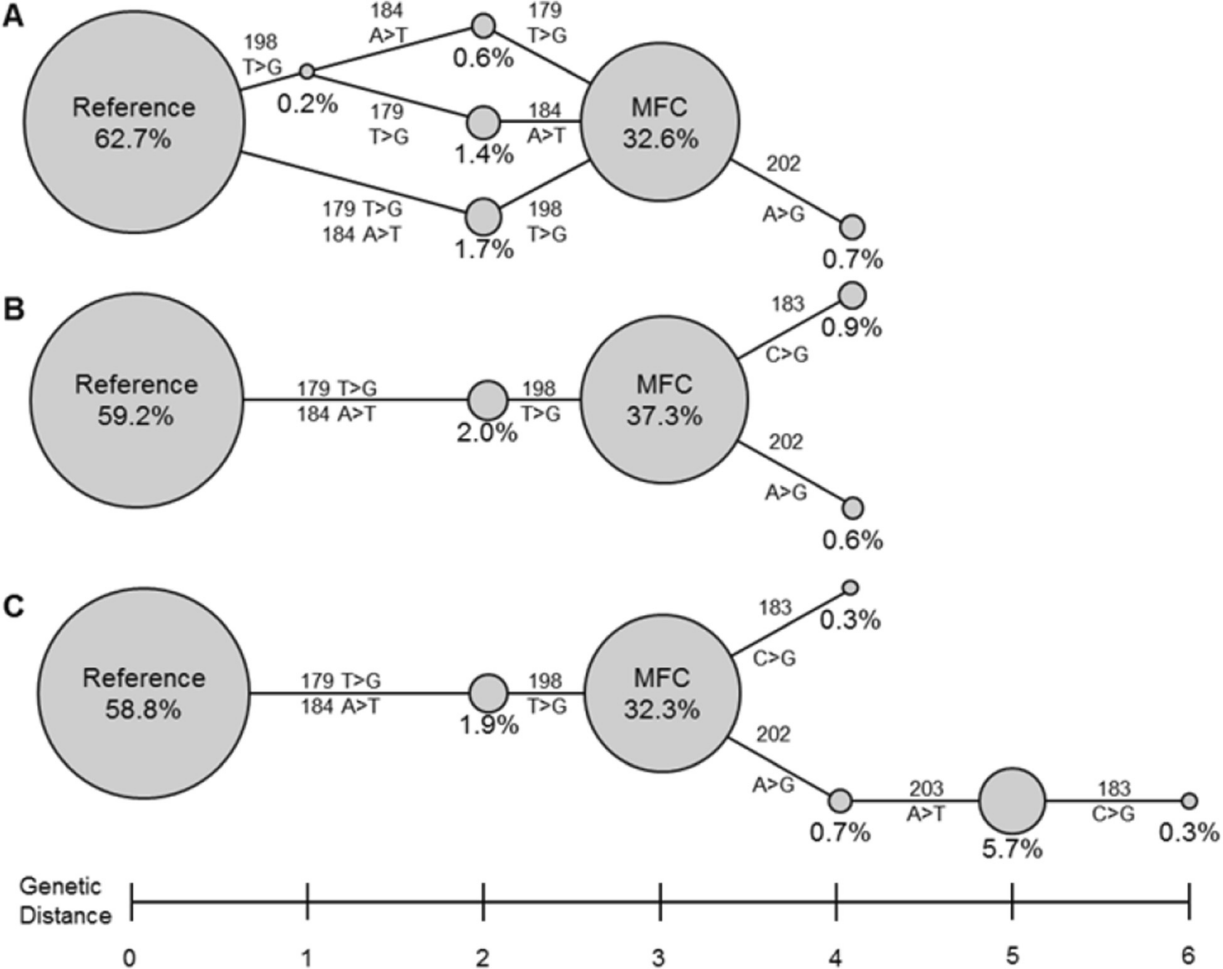
**High resolution dendrograms generated from iterated ROA dictionaries provide historical perspective on tumor subclone development.** These dendrograms from verified words in FL-128 *BCL2* ROA 171 to 204 dictionary depict an inferred developmental relationship between putative tumor subclones, with nodes depicting observed verified word sequences and lines representing mutational differences between words. The relative population fraction is reflected by node circle area and genetic distance from the normal reference sequence by horizontal displacement. Mutations are noted by position and called SNV. **(A)** Seven clones are found in specimen 128 using BFAST mapping without iteration in which an ambiguous developmental history of the most frequent clone (MFC) is seen along with a single descendant. Mapped coverage is 14,623 reads. **(B)** Five clones are found in specimen 128 by iterating BFAST to convergence (4 mapping iterations) in which the most frequent clone has two low frequency descendants. Mapped coverage is 15,492 reads. **(C)** Seven clones are found in specimen 128 by mapping first with BFAST, followed by SHRiMP2, and then iterating BFAST to convergence (BSBn method, 4 mapping iterations) in which a 5.7% clone and its 0.3% descendant containing mutations at two adjacent positions (202 and 203) were revealed by SHRiMP2. Mapped coverage is 15,594 reads.

Phylogenic analysis of regional sequence variation obtained from ROA dictionary output, coupled with word frequency data, provide an historical perspective on tumor subclone development, as well as a minimal estimate of tumor heterogeneity. Both iteration and use of alternate mapping algorithms bring clarity and enrich the complexity of dendrograms from a region with significant mutations, as seen in FL-128 *BCL2* ROA 171 to 204 (Figure 10). DDiMAP analysis of single round BFAST mapping, fully iterated BFAST mapping and alternating mapping with both BFAST and SHRiMP2, followed by BFAST to convergence all found three base changes between human genome reference sequence and the MFC in this region, along with continued evolution from the MFC. The dictionary from a single round of BFAST mapping generated an ambiguous dendrogram for this population, as four different ‘putative ancestral’ word sequences to the most frequent clone were observed, denoted as nodes connected by lines identified by the mutational difference between words (Figure 10A). Iteration to convergence using BFAST alone allowed these apparently parallel mutation pathways to coalesce into a single ancestral tree, bringing clarity to the developmental pathway (Figure 10B). Equally striking is the effect of BSB_n_, through the recognition of a mutation doublet by SHRiMP2 in the second iteration, identifying a new descendant branch off the MFC that occurs at a much higher proportion than the other descendants, suggesting either an earlier occurrence or a relatively faster growth rate of this subclone (Figure 10C). While word sequence and frequency analysis provide an inferred development pathway, it is important to note that not all mutational events may be observed, as reversion to reference sequence is possible in mutational hot-spots, and if a specific base change generates a mutational motif, the probability of additional mutations at that location will be increased.

## Conclusions

The design goal for DDiMAP is to evaluate tumor heterogeneity in follicular lymphoma, a cancer of B-lymphocytes that is well characterized with regard to ongoing AID-mediated somatic hypermutation of *IGH*, with the long-term objective of classifying FL based on their mutational patterns. We found that *BCL2*, followed by *BCL6*, showed the most consistent and differential aSHM signal in FL specimens, with SNVs in 12/12 FL cases and 0/3 in the reactive LN controls. The range of SNVs in *BCL2* varied widely, from 2 to 101 per patient; raising the possibility that overall *BCL2* aSHM rate might be clinically informative through risk stratification [54]. *BCL6* shows a similar pattern, but with a much lower overall aSHM rate, in the range of 2 to 39 SNV per patient. No other genetic regions evaluated had either the consistency or differentiation potential of these two gene regions (see Additional file 4: Table AF1). We evaluated the SNV patterns in *BCL2* to investigate the distribution of SNV frequencies relative to the MFC. The frequencies associated with MFC varied from 10% to 45% (median of 20%) and in all but one case (FL-134) the majority of SNVs in any given tumor had frequencies consistent with their current MFC. Several FL specimens (5/12) had no SNVs outside the MFC; three with a low overall mutation rate (<0.7%) and two with a different type of FL (Grade 3 vs. Grades 1 and 2), suggestive of a possible biological aspect to this finding [54]. Of the remaining FL specimens, the vast majority (over 80%) of the SNVs outside the MFC were found at ≤3%, while over half were ≤1% (see Additional file 4: Table AF1). This emphasizes the need for robust identification of SNVs at these levels to detect tumor heterogeneity, and that previous estimations of diversity may be artificially low due to technical limitations [48].

Maintaining the variant calls as words is the key component enabling many aspects of DDiMAP. First, it can be used as an alternate approach to model-free filtering of instrumental noise by taking advantage of the correlation of true mutations along an allele while eliminating the high level of uncorrelated noise typical of massively parallel sequence data. Second, these regional allele specific words can be used to objectively identify and quantify tumor subpopulations, enabling regional genetic sequence assembly to describe the development of tumor subpopulations on a fine scale, an illuminating view of subpopulation dynamics. Additionally, assembled overlapping words can be used to augment reference sequence files for iterative remapping.

While iterative remapping is not an essential component of DDiMAP, it is critical to achieve adequate mapping of reads from regions with dense mutation loads. A major concern with iteration was the possibility of generating false SNV calls, especially at low frequencies. We have several lines of evidence this did not occur, including multiple genetic regions from all patient samples that were found to be free of SNV calls following multiple rounds of iteration and a consistent bias in both high and low frequency SNVs to occur at an AID motif, indicating that both represent a biological process. Additionally, in the vast majority of cases, the SNV calls made within a ROA were internally consistent with an evolutionary progression of mutations, allowing the development of dendrograms utilizing all verified reads. This would not be the case if low level mutations were randomly generated false calls.

Mutational hot spots, due to aSHM or other causes of katageis, are prime regions for analysis of tumor heterogeneity and determination of ongoing mutation. We identified problems with aligning reads to these regions as a significant limiting factor in SNV analysis of these areas and that mapping failure was found in both SOLiD and Illumina platforms using multiple aligners. Additionally, the majority of subpopulations are defined by SNVs present at low frequencies (<3% in FL) requiring highly sensitive variant detection in these problematic areas. DDiMAP, based on analysis of variant sequence patterns, provides the necessary tools overcome both these problems: partial sequence assembly for both noise reduction and SNV calling, and generation of additional allele fragments for iterative remapping. While we have evaluated this approach for its utility in cancer research, it readily applicable in any mixed population analysis distinguished by clustered changes in genomes.

## Additional files

Additional file 1:
**Enhanced Methodology: is an enhanced methodology section which includes details on DDiMAP adjustable parameters, SNV calling, simulated data generation, and sample preparation information.**
Additional file 2:
**DDiMAP User Guide: is a User Guide for the sample software [** 35 **].**
Additional file 3:
**Reference sequences: contains the reference sequences, including Sanger sequence of the specimen specific** ***IGH*** **.**
Additional file 4:
**Supplemental Data: contains supplemental data tables and figures.**
Additional file 5:
**SNP analysis: contains worksheets listing all SNPs detected in the FL-SOLiD data.**

## Abbreviations

(a)SHM: (aberrant) Somatic hypermutation
AID: Activation induced cytidine deaminase
CDR1-3: Complementarity determining regions within *IGHV*
DDiMAP: Deep-drilling with iterative mapping
FL: Follicular lymphoma
FPR: False positive rate
FR1-3: Framework regions within *IGHV*
*IGH*: Immunoglobulin heavy chain
Indel: Insertion/deletion
*IGHV*: IGH Variable region
MFC: Most frequent clone
NGS: Next generation sequencing
PCR: Polymerase chain reaction
PPV: Positive predictive value
ROA: Region of analysis
SNV: Single nucleotide variation
VAF: Variant allele frequency
