# Supplementary material for: Ultradeep analysis of tumor heterogeneity in regions of somatic hypermutation
Source: Genome Med. 2015 Mar 12;7(1):24. doi: 10.1186/s13073-015-0147-1 (PMC4395979; doi:10.1186/s13073-015-0147-1)
Supplement: Additional file 1: — Enhanced Methodology: is an enhanced methodology section which includes details on DDiMAP adjustable parameters, SNV calling, simulated data generation, and sample preparation information. [file 13073_2015_147_MOESM1_ESM.pdf]

Methods

Use of Multiple ROA Collections

Coverage irregularities are common in massively parallel sequence data and differences in coverage may lead to significant differences in the read distribution for one start position compared to another nearby position. When this distribution of reads across overlapping ROAs highly favor one side, a true variant may be eliminated by the two-step filter solely due to a lack of coverage in one of the overlapping ROAs. One solution is to generate a second ROA collection with an offset start location that will result in both building ROA dictionaries using a different partitioning of the reads and using differing portions of each read in dictionary formation (Fig. 1). Identifying variant candidates across multiple ROA collections allows detection of variants that are missed when using a single ROA collection. If more than one start position is to be considered, for computational efficiency, ROA dictionary collections for all possible start positions may be formed in a single pass through the mapped read data file, from which an ROA dictionary collection for any number of start positions may be selected for analysis. It is not necessary to look at results from all possible start positions to effectively enhance sensitivity and a dual start position analysis with an offset between them equal to half of the overlap length often suffices. For each alternate start position ROA word collections, we tabulate the nucleotide data at each position of the reference sequence from the words in the dictionaries according to their verification status and ROA start position and compute frequencies of each base at each location relative to the local coverage.

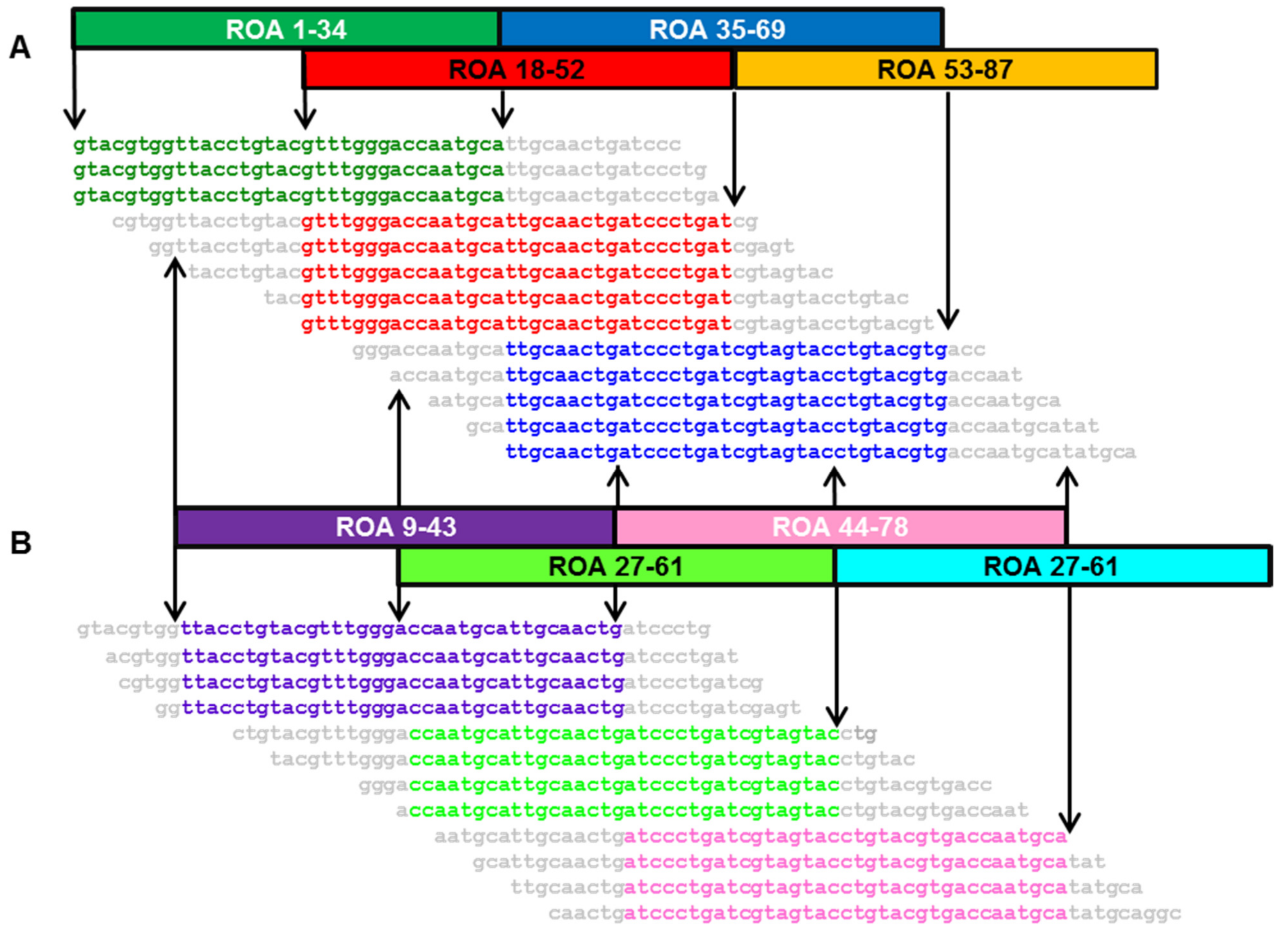

Figure 1. Multiple ROA Collections and Read Assignment.

The reference sequence is divided into ROAs starting at a set position, according to chosen ROA size and desired overlap. Reads are assigned to ROAs according to bam alignment information, based on the read sequence completely covering the ROA endpoints, with trimming of excess sequence (greyed bases). **(A)** This diagram represents an ROA Collection with 2-tracks of abutting ROAs of size 34 bases with a second track that overlaps the first by one-half (17 bases). The reads are uniquely assigned to one track such that abutting and overlapping ROAs from a single collection contain independent sets of reads. In this case, 50 base reads are divided into ROAs of 34 bases with 17 base overlap. **(B)** This diagram represents a second ROA Collection with a start site of 9 which is offset from the first collection by one-half the overlap. The sequence differences in read sets generated by this alternate partitioning are denoted by vertical arrows. Reassigning the reads into different ROA collections increases the ability to confirm variants in regions with severe differences in coverage.

Candidate Variant Identification

The final stage identifies candidate variants by applying three simple rules:

1) A candidate variant is identified if it is present in a fully verified word in both ROA collections, regardless of its frequency,

2) A candidate variant is identified if it is present in a fully verified word in either ROA collection at a sufficiently high frequency, and

3) A candidate variant may be identified if it is present at a much higher frequency even if the word(s) containing the call are not fully verified. However, such candidates require careful scrutiny, especially with variants near ends of the ROA.

The threshold frequency for type 2 calls is typically chosen at four times the frequency used for dictionary formation and the threshold frequency for type 3 calls is typically chosen at 10% or higher and cautiously used only at boundaries of extremely low coverage regions.

Shared Variant Removal

After gathering the identified variant candidates, we classify as shared variants all identical variants that occur at the same approximate low frequency in any controls and test specimens as probable library and/or polymerase generated sequence errors (shared events) and manually remove these data from further consideration.

Considerations for ROA Size Selection

ROA size needs to be chosen in a way that is easily programmed, linked to the read size for efficient read utilization, and when iteration is used, tied to the assembly method used for iteration. An ROA size that is even makes overlapping ROA analysis simpler to program. An ROA size longer than 2/3 of the read length will result in some reads

not being assignable to an ROA in a single ROA collection. If multiple collections are used, reads that are incompatible in one collection may be compatible in another and vice versa. The assembly procedure we employ performs a single half word extension in each direction around a core word, resulting in a fragment twice the length of an ROA. For such a fragment to be useful for mapping, it needs to be at least as long as a (trimmed) read, so the ROA length needs to be at least half the (trimmed) read length. Additional length is preferred to permit mapping of reads at multiple start locations within the fragment. The shortest length that results in a collection of alternate allele fragments that permits mapping of any read depends upon the number of collections that are employed. This length can be expressed as a fraction of the read length which depends on the number of collections N as 2*N/(4*N-1), which is 2/3 for a single collection, 4/7 for a dual collection and so on. For a read length of 50bp using the simple assembler and dual ROA collections, the minimal size is the first even number in excess of 200/7, which is 30. Use of this minimal size will result in the final 5 bases of any read never being utilized. The longest that allows for analysis of all but a few reads is 34, just over 2/3 of the read length. Use of this size will result in utilization of the final bases depending on the start position of the ROA collection and the mapped location range of the read. When doing final analysis, the minimal length constraint may be relaxed as no further assembly for mapping is required, but the power of the cross-verification filter is diminished if the ROA length becomes shorter, so use of shorter ROA sizes typically requires use of higher thresholds to avoid false positive variant identification.

Considerations for aligner selection

We evaluated several commonly used aligners at their default settings, BFAST 0.7.0a, SHRiMP2 2.3.0, CUSHAW3, BWA-MEM, and Novoalign, for effects on DDiMAP performance. Global alignment, which maps the entire read, tends to generate false variants from gapping used to match both ends of reads to the reference. These tend to show as type 3 calls that are non-verified but at high frequency. We use type 3 calls from global aligners only for generation of additional alleles to allow for alternate reference growth into low coverage areas but not for final variant calls. Local alignment has different behavior. Use of soft clipping may lead to loss of coverage in areas with high divergence relative to the reference as the effective shortening of the read may cause it to fail to cover any ROA. In their default configurations, BFAST and SHRiMP2 use global alignment whereas CUSHAW3, BWA-MEM, and Novoalign permit local alignment. While the latter three use soft clipping, CUSHAW3 has robust algorithms that allow recovery of coverage at the cost of some level of false positives while BWA-MEM and Novoalign are more stringent, resulting in high PPV but failing to grow coverage across regions of dense variation. Additionally, Novoalign uses IUPAC "N" codes for locations at which it is uncertain of interpretation of the read. Our test code rejects all reads containing "N", leading to a loss of coverage.

Execution time for a single round of DDiMAP dictionary formation, variant identification, and alternate allele assembly using our test code is small compared to the time taken by mapping. In our implementation, over 90% of the time is spent in bam file reading. Mapping time varies widely across the mappers we used, with newer algorithms providing significant performance enhancement, enabling iteration to

completion in a matter of minutes rather than hours or even days. The efficient algorithms in CUSHAW3 and BWA-MEM result in the shortest iteration times among the mappers we employed.

Simulation Experiment

Sanger sequence data containing a rearranged *IGH* gene from a FL specimen was chosen to represent a founder clone of length 1864 nt. Ongoing mutation was simulated using ten generations of duplication and mutation wherein locations were changed randomly at a 1/10000 rate after each duplication. This results in 1024 full length sequences containing ongoing mutations at frequencies as low as 1/1024. A random fragment library with a 200 nt average size and 5.5% CV was generated from these full length sequences using the simLibrary routine [1], with 1000 fragments generated from each sequence. A set of 100 nt simulated reads was generated from these fragments using the simNGS routine [1] and a runfile [2] describing the variance/covariance structure of read errors from a 100 nt paired end run of an Illumina HiSeq machine. The resulting read and quality data formed a fastq file which was used as input for the mappers.

The *IGH* Sanger sequence data for this FL specimen was interpreted by IMGT/Vquest [3] as a productive rearragement of *IGHV*4-61 and *IGHJ*5 with a 42 base junctional region. The germline reference sequences used for mapping include an *IGHV*4-61 sequence starting 84 nt upstream of FR1 and ending 8 nt beyond the end of FR3 together with *IGHJ* sequence encompassing *IGHJ*1 through *IGHJ*6. No reference sequence is provided for the non-templated junction between the *IGHV* and *IGHJ* sequences, so reads extending into this region are typically not well mapped.

Iteration was performed using DDiMAP with an ROA size of 58, a 1% threshold for inclusion of fragments built around verified core words, and a 10% threshold for inclusion of fragments built around non-verified core words. Variant identification was performed using an ROA size of 64 nt and thresholds varying over a range from 100ppm to 800ppm to obtain data for precision-recall curves. Data is selected from the FR1-FR3 locations of the *IGHV* reference sequence.

Pooled Sample Experiment

A pooled sample experiment is used to assess performance of the DDiMAP variant identification procedure from a given set of mapped read data and demonstrate its capability to discriminate multiple allele patterns. SOLiD 50 base fastq read data was mapped using BFAST to obtain a set of mapped read bam files for each of 12 FL specimens, 8-10 M reads per specimen. The bam files were separately preprocessed using SAMTOOLS mpileup [4] and analyzed with VarScan2 using its mpileup2snv command at a threshold setting of 1% to define a collection of gold standard variants for each specimen from the *BCL2* gene. The bam files were then pooled into a single file, preprocessed using mpileup and analyzed by VarScan2 at threshold settings from 0.1% to 1.0%. The pooled bam file was also analyzed by DDiMAP at primary threshold settings from 100ppm to 800ppm and an ROA size of 34. The gold-standard variant calls were validated by Sanger sequencing where possible, and all Sanger level SNV calls were included in the standard. Additionally, as VarScan2 only reports the most frequent variant at any location, lower frequency variants from locations at which multiple variants were present in the pooled sample are not included in compiling data for precision-recall analysis.

In order to derive a logistic model for DDiMAP sensitivity as a function of the underlying variant frequency, nominal pooled frequencies are required along with presence/absence data for each variant at a specific primary threshold. For this purpose, frequencies reported for the gold standard variants in the pooled data using VarScan2 are used when available. For lower frequency variants at locations with multiple variant calls and therefore missed by VarScan2, a pooled frequency estimate is obtained by applying the coverage ratio of the missing variant to a nearby variant from the corresponding specimen to the nominal frequency of the missing variant. Logistic regression is then performed using mnrfit from the MATLAB Statistics toolbox [5] using the logarithm of the nominal pooled frequency as the predictor input variable. Predicted detection probability estimates and 95% confidence regions for the predictions as a function of frequency are obtained using the MATLAB mnrval function.

Cross-blending experiment

A second blending experiment is used to more tightly characterize the sensitivity vs frequency performance of DDiMAP using the threshold chosen for the FL SOLiD results (Figure 7). In this experiment, individual reads are randomly selected from fastq files from two specimens with differing *BCL2* mutation patterns such that the total number of reads in each blend is held constant (10 M), but the amount of data from each specimen varies from 1/32 to 31/32 of the blended read pool. Variants used for sensitivity analysis are those detected at a 3% or higher level by DDiMAP using a frequency threshold of 750ppm and an ROA size of 34 in a bam file created using a single round of BFAST applied to their pure specimen data. Nominal blended frequencies are obtained by scaling the pure specimen frequency by the specimen proportion in each

mixture. The blended read pools are mapped using a single round of BFAST to obtain bam files that are separately analyzed using DDiMAP at the same threshold and ROA size. Presence/absence data were aggregated from the various analyses and fit to a logistic model as done in the pooled sample experiment.

Supplemental Methods: DNA preparation and Amplicon production

SOLiD NGS

The SOLiD data used to develop this analytical pipeline was from a PCR based targeted re-sequencing of upstream noncoding regions of selected genes from follicular lymphoma (FL) specimens, a B cell tumor that has been shown to have ongoing AID activity through continued SHM within *IGH [6-9].* Test specimens include lymph node biopsies from 12 FL tumors, 3 hyperplastic lymph nodes (HP) as a source of non-malignant polyclonal B cells, all obtained as de-identified samples from the Human Hematological Malignancy Tissue Bank at URMC in accordance with institutional IRB protocols, and HEK 293 as a source of clonal non-lymphoid tissue.

Genetic targets were selected for a variety of reasons, including published findings of aberrant SHM in DLBCL and FL in humans (proto-oncogenes *BCL6*, *PIM1*, *PAX5*, *RHOH*, and *MYC [10-12]* and in DNA repair deficient mice (*CD83* and *SYK*) [13]. *BCL2*, a component of the hallmark t(14;18) genetic lesion of FL, was included because of its high rate of expression, a critical requirement for aberrant SHM. Two *IGH* regions, the natural targets of SHM, were also studied, including an *IGHJ* intronic region (*IGHJ*-enh) common to all cells as well as the tumor specific rearranged *IGH* from the FL specimens. See Table 1 for a complete list of primer locations and sequences.

DNA Preparation for all specimens

Single cell suspensions were prepared from lymph nodes and viably frozen by the Human Hematological Malignancy Tissue Bank at URMC without any cell selection. DNA was extracted from ~5e^6 cells using the QIAamp DNA Mini Kit (Qiagen Inc., Valencia, CA) according to standard protocol and the concentration was estimated by spectrophotometry (NanoDrop, Wilmington, DE).

NGS Amplicon generation

The PCR was performed according to manufacturer instructions with Phusion hot start high fidelity DNA polymerase (NEB, Ipswich, MA) using HF buffer and 3% DMSO. Template amount (250 ng) was chosen to ensure sampling of sufficient cells (~40,000) for statistically valid estimation of low frequency events, designed to be sufficient for identification of a 0.1% population at >95% probability in the absence of instrumental error. The reaction was cycled 35 times between 98°C for 10 seconds, 66-72° C for 15 seconds and 72°C for 45 seconds, preceded by 3 minutes at 98°C, and followed by 5 minutes at 72°C. Stringent annealing temperatures were chosen to enhance primer specificity and limit off target binding. Amplicons were screened for correct size by electrophoresis on 0.7% agarose gels in TAE buffer (Invitrogen/Life Technologies, Grand Island, NY), purified with QIAquick PCR Cleanup kit (Qiagen Inc.) and quantified by spectrophotometry (NanoDrop).

***IGH* Identification**

IGH was amplified from the FL specimens using the Biomed 2 IGH framework 2 primer set and JH consensus primers [14]. Clonal IGH was identified by heteroduplex analysis of the PCR amplicons, followed by gel purification and sequencing of homoduplex bands. The Biomed 2 PCR reaction used 50 ng of genomic DNA as template with

HotStarTaq (Qiagen) according to manufacturer directions. The reactions were cycled 35 times between 94°C for 30 seconds, 61° C for 30 seconds and 72°C for 90 seconds, preceded by 5 minutes at 94°C, and followed by 5 minutes at 72°C. Heteroduplex analysis was performed by heating the amplicons for 5 minutes at 98°C, followed by rapid cooling to 4°C for 2 hours. Homoduplexed DNA was identified using a 2% NuSieve 3:1 agarose gel (Lonza Basel, Switzerland), purified using QiaexII gel purification kit (Qiagen) according to manufacturer directions and sequenced. *IGHV* used for each FL specimen was identified using IgBLAST [15] or V-Quest [3]and the final amplicon for SOLiD sequencing was generated using gene-specific *IGHV* primer paired with a common *IGHJ*-region primer (Pv235) located downstream of *IGHJ*6, using the PCR parameters as described for NGS amplicon generation above.

pBluescript II KS negative control (Agilent Technologies, Inc., Santa Clara, CA)

A purified plasmid preparation of pBluescript II KS was digested with *Pvu*I (NEB) according to manufacturer protocol. The 1045 nt fragment was isolated using a 0.7% agarose gel (Invitrogen/Life Technologies, Grand Island, NY), purified with QIAquick Gel Extraction kit and the concentration was estimated by spectrophotometry (NanoDrop).

Library preparation and SOLiD sequencing.

For each specimen, the amplicons were mixed in equimolar ratios, FL specimens were spiked with 0.1 equimolar pBluescript II KS fragment, and 2 μg of mixed amplicons were submitted to the Genomics Research Center at the University of Rochester for library preparation and SOLiD 4 sequencing. As part of the library preparation, the amplicons were concatenated, fragmented with the Covaris S2, and appropriately barcoded according to ABI standard protocols.

HL *IGH* amplicon for MiSEQ NGS

IGH amplicons were generated from 100 ng gDNA using a multiplexed PCR reaction consisting of 3 master mixes (P1, P3 and P4) with primers designed to amplify all functional *IGHV* genes coupled with a single downstream *IGHJ* primer. See Table 1 for primer location and sequence.

The PCR reaction used 100 ng of genomic DNA as template with HotStarTaq (Qiagen) according to manufacturer directions. The reactions were cycled 35 times between 94°C for 30 seconds, 61° C for 30 seconds and 72°C for 4 minutes, preceded by 5 minutes at 94°C, and followed by 10 minutes at 72°C. The PCR products were purified using QIAquick PCR clean-up kit, blunted and phosphorylated with NEB quick blunt kit and concatenated with T4 ligase (NEB), all according to manufacturer's directions. The MiSeq library was prepared using the Illumina Nextera kit. The MiSeq library preparation and sequencing run were performed by the Genomics Core Facility at Cornell Center for Comparative and Population Genomics (Ithaca, NY)

Table 1. Listing of primers used to amplify genetic regions of interest.

Gene	Chr	Primer	Chromosome Location*	Sequence	Size (bases)
BLC2	18q21	Pv295	18:60986652	5' GCTCTTGAGATCTCCGGTTGGGATTCC 3'	1078
		Pv265	18:60985575	5' GGTAGCGGCGGGAGAAGTCGTCG 3'	
BCL6	3q27	Pv239	3:187463235	5' GGTGATGCAAGAAGTTTCTAGGAAAGG 3'	948
		Pv238	3:187462288	5' CGGCTCTCATTAGGAAGATCACG 3'	
CD83	6p23	Pv299	6:14117875	5' CCCCGGCCTAAGCGGGACTAGGAG 3'	1558
		Pv302	6:14119432	5' CAGAGCACCTTTGCAATTTATAGGG 3'	
IGH-enh	14q32	Pv259	14:106328444	5' GCCACCTGCTGTGGGTGCCGGAGAC 3'	951
		Pv275	14:106329394	5' CTCTAGGGCCTTTGTTTTCTGCTACTG 3'	
MYC TSS-1	8q24	Pv244	8:128748420	5' AAAGAACGGAGGGAGGGATCG 3'	1104
		Pv247	8:128749532	5' ACCCAACACCACGTCCTAACACCT 3'	
MYC TSS-2	8q24	Pv246	8:128750451	5' CTTTAACTCAAGACTGCCTCCCG 3'	660
		Pv245	8:128751110	5' TCGTTGAGAGGGTAGGGGAAGAC 3'	
PAX5	9p13	Pv248	9:37026233	5' TACGGGTCCAAGCCAGGGTTCTCC 3'	888
		Pv249	9:37027120	5' GCCCCAGAGACTCGGAGAAGCAGA 3'	

PIM1	6p21	Pv241	6:37138166	5’ CAGCGCCCTCAGTTGTCCTCCG 3’	1106
		Pv240	6:37139271	5’ TCACCATCGAAGTCCGTGTAGAC 3’	
RHOH	4p13	Pv243	4:40198904	5’ TCGGCATTCTGCAACAGGTAAGG 3’	971
		Pv242	4:40199874	5’ CCTTCTCCTTCTACCGACACTTC 3’	
SYK	9q22	Pv290	9:93564046	5’ GCGTTAAGGAAGTTGCCCAAAATGAG 3’	1098
		Pv292	9:93565143	5’ CCAAGAATCAGATATGGCACAACATC 3’	
IGHJ	14q32	Pv235	14:106329305	5’ CGCCCAGGTCCCCTCGGAACATGCC 3’	
*IGHV*3-48	14q32	Pv272	14:106994331	5’ GCCTTAGCCCTGGATTCCAAGGCATT 3’	
*IGHV*1-18	14q32	Pv276	14:106642227	5’ GGTAGGGGATGCGTGGCCTCTAAC 3’	
*IGHV*4-39	14q32	Pv280	14:106878110	5’ GTCCAACTCATAAGGGAAATGCTTTCTG 3’	
*IGHV*1-8	14q32	Pv282	14:106539605	5’ GGTAAATATAGGTATATTGGTGCCCTG 3’	
*IGHV*1-46	14q32	Pv273	14:106967471	5’ GAGGGTCTTCTGCTTGCTGGCTGTAGC 3’	
*IGHV*3-7	14q32	Pv274	14:106518864	5’ GTCTCAGAGAGGAGCCTTAGCCCTGGACTC 3’	
IGHV4 family	14q32	Pv251	many sites	5’ CCCAGATGGGTCCTGTCCCAGGTGCAG 3’	
IGHV3 family	14q32	Pv252	many sites	5’ AAGGTGTCCAGTGTGAGGTGCAG 3’	
IGHJ family	14q32	Pv30	many sites	5’ ACCTGAGGAGACGGTGACCAGGG 3’	
IGHJ3	14q32	Pv31	many sites	5’ ACCTGAAGAGACGGTGACCATTGTC 3’	
IGHJ family	14q32	Pv32	many sites	5’ ACCTGAGGAGACAGTGACCAGGG 3’	
IGHJ6	14q32	Pv33	many sites	5’ CTTACCTGAGGAGACGGTGACCGTG 3’	
P1	14q32	Pv367	many sites	5’ ATGGACTGGACCTGGAGCATCCTCTTCTTGGTGG 3’	
	14q32	Pv385	many sites	5’ GTCATTCTCTACTGTGTCCTCTCCGCAGGTGCTCACTCCC 3’	
	14q32	Pv378	many sites	5’ CCTCGCCCTCCTCCTGGCTGTTCTCC 3’	
	14q32	Pv489	many sites	5’ CACTGGGCTGAGGGAGAAACCAGCAC 3’	
P3	14q32	Pv383	many sites	5’ ATGGAGTTGGGGCTGAGCTGGGTTTTCC 3’	
	14q32	Pv382	many sites	5’ GAAACAGTGGATACGTGTGGCAGTTTCTGAC 3’	
	14q32	Pv374	many sites	5’ GAAACAGTGGATTTGTGTGGCAGTTTCTGAC 3’	
	14q32	Pv384	many sites	5’ TTGTCTCCTTTGTGGGCTTCATCTTCTTATG 3’	
P4	14q32	Pv380	many sites	5’ ATGAAACACCTGTGGTTCTTCCTCCTGCTG 3’	
	14q32	Pv381	many sites	5’ ATGAAACACCTGTGGTTCTTCCTCCTCCTG 3’	
	14q32	Pv379	many sites	5’ CTGGTGGCAGCTCCCAGATGTGAGTATCTC 3’	
	14q32	Pv376	many sites	5’ ATGTCTGTCTCCTTCCTCATCTTCCTGC 3’	

*Location according to GRCh37.p13 at www.NCBI.NLM.NIH.gov, accessed 10/09/2013

1. [https://github.com/timmassingham/simNGS]
2. [http://www.ebi.ac.uk/goldman-srv/simNGS/runfiles5/HiSeq/s_1_4x.runfile]
3. [http://www.imgt.org/IMGT_vquest/vquest?livret=0&Option=humanIg]
4. [http://samtools.sourceforge.net/mpileup.shtml]
5. [http://www.mathworks.com]
6. Zelenetz AD, Chen TT, Levy R: **Clonal expansion in follicular lymphoma occurs subsequent to antigenic selection.** *J Exp Med* 1992, **176:**1137-1148.
7. Bahler DW, Campbell MJ, Hart S, Miller RA, Levy S, Levy R: **Ig VH gene expression among human follicular lymphomas.** *Blood* 1991, **78:**1561-1568.
8. Zhu D, Hawkins RE, Hamblin TJ, Stevenson FK: **Clonal history of a human follicular lymphoma as revealed in the immunoglobulin variable region genes.** *Br J Haematol* 1994, **86:**505-512.

9. Ottensmeier CH, Thompsett AR, Zhu D, Wilkins BS, Sweetenham JW, Stevenson FK: **Analysis of VH Genes in Follicular and Diffuse Lymphoma Shows Ongoing Somatic Mutation and Multiple Isotype Transcripts in Early Disease With Changes During Disease Progression.** *Blood* 1998, **91:**4292-4299.
10. Halldórsdóttir A, Frühwirth M, Deutsch A, Aigelsreiter A, Beham-Schmid C, Agnarsson B, Neumeister P, Richard Burack W: **Quantifying the role of aberrant somatic hypermutation in transformation of follicular lymphoma.** *Leukemia research* 2008, **32:**1015-1021.
11. Pasqualucci L, Neri A, Baldini L, Dalla-Favera R, Migliazza A: **BCL-6 mutations are associated with immunoglobulin variable heavy chain mutations in B-cell chronic lymphocytic leukemia.** *Cancer Research* 2000, **60:**5644-5648.
12. Pasqualucci L, Neumeister P, Goossens T, Nanjangud G, Chaganti RS, Kuppers R, Dalla-Favera R: **Hypermutation of multiple proto-oncogenes in B-cell diffuse large-cell lymphomas.** *Nature* 2001, **412:**341-346.
13. Liu M, Duke J, Richter D, Vinuesa C, Goodnow C, Kleinstein S, Schatz D: **Two levels of protection for the B cell genome during somatic hypermutation.** *Nature* 2008, **451:**841-845.
14. van Dongen JJM, Langerak AW, Bruggemann M, Evans PAS, Hummel M, Lavender FL, Delabesse E, Davi F, Schuuring E, Garcia-Sanz R, et al: **Design and standardization of PCR primers and protocols for detection of clonal immunoglobulin and T-cell receptor gene recombinations in suspect lymphoproliferations: Report of the BIOMED-2 Concerted Action BMH4-CT98-3936.** *Leukemia* 2003, **17:**2257-2317.
15. [http://www.ncbi.nlm.nih.gov/igblast/]
