# Supplementary material for: Ultradeep analysis of tumor heterogeneity in regions of somatic hypermutation
Source: Genome Med. 2015 Mar 12;7(1):24. doi: 10.1186/s13073-015-0147-1 (PMC4395979; doi:10.1186/s13073-015-0147-1)
Supplement: Additional file 2: — DDiMAP User Guide: is a User Guide for the sample software [ 35 ]. [file 13073_2015_147_MOESM2_ESM.pdf]

DDiMAP and DDiMapIterate.py User Guide

System Requirements:

Your system should be capable of running a NGS mapper such as bwa, SHRiMP, BFAST, Novoalign, CUSHAW3, or the like. We are able to run on a Linux or Mac workstation class machine with a multicore 64 bit processor and 2GB RAM per core for datasets containing 10M reads and an approximately 100Kbp reference sequence. The major memory footprint is driven by the mapper and the target reference sequence size, with DDiMAP analysis typically having a lower memory footprint.

Installation of DDiMAP and Associated Software

Required software and packages:

Compiler: gcc 4.8 or higher, cmake; these are commonly installed, to see if they are present and the version info, try the commands: "gcc --version" and "cmake /V" to see what you have

Scripting language: Python 2; it is likely already installed on your system, try the command "python --V" to see what version you have

Helper tools: BioPython 1.65 or later, possibly installed on your system.

DDiMAP and DDiMapIterate.py installation:

In a command shell, cd into a directory that will contain the software such as your $HOME/bin or another directory in which you have write access.

Get the software from github using the command

git clone https://github.com/jpspence/DDiMAPpublic.git

This will create a directory DDiMAPpublic. You can change the name of the directory. Then cd into the directory and make the software. The "make setup" command will get the supporting C/C++ packages needed by DDiMAP, including bamtools. The "make" command will compile the code and create the DDiMAP executable in the bin subdirectory.

Using DDiMAP and DDiMapIterate.py

Configuration File

A sample configuration file is provided in the bin directory (DDiMap.cfg) and in each of the example directories in the data directory .. Such a file may be used to completely specify all parameters for use by the DDiMapIterate.py script, also found in the bin directory, which fully implements the iterative workflow shown in Figure 1 of the paper.

A single pass of mapping using a configured mapper and DDiMAP analysis may be run by setting the maximum number of iterations to 1.

Reference Sequence Files

Reference sequences must be in fasta format, containing all sequences to use for mapping the read data. Multiple reference sequence files may be provided and will be combined into a single reference sequence file for use by the mapper. The identifiers for each of the sequences in your provided files must not contain underscore "_" characters: use a hyphen "-" instead as DDiMapIterate.py reserves the underscore characters to construct reference sequence IDs for use by DDiMAP.

Read Data Files

Read data files are typically provided in fastq format for use by the mapper routine along with reference sequence file(s). Single end read data is always in a single file. Mappers vary in how they expect to see paired-end data presented. A pair of files is most common, but some require interleaving of read data into a single file. DDiMapIterate.py can be configured to deal with either, but the currently supported mapper configurations are all of the two file variety. See "How to Add a New Mapper" below for details.

Output Files

Each run uses a single specified output directory to contain intermediate results in separate subdirectories for each iteration and final results, including comma separated value (csv) formatted files containing coverage data, dictionary data, and variant data along with fasta formatted files containing alternate reference sequence fragments generated by DDiMAP and the complete set of reference sequences used for the final mapping and analysis iteration. Each of the intermediate result directories contain these files for that particular iteration as well as log files for the mapper/samtools/DDiMap steps of the process for error tracing. Currently, other files are kept in these directories such as mapper indexes and the aligned read bam files. Additionally, a copy of the configuration file used (including the selected settings used for analysis) is provided in the main output directory as a record.

Command Line Interfaces and Configuration File

DDiMAP may be run directly using a command line interface against a fasta/bam file combination resulting from running a mapper on a fasta/fastq file combination. However, the DDiMAP program uses specific patterns in the reference sequence names (see the discussion of the Enhanced Reference Sequence File below) and spacing in the file (only single line between reference sequences) that are enforced by the DDiMapIterate.py script. It will not run correctly if you use a noncompliant reference sequence file. Sample MATLAB scripts that implement an alternate interface are also provided in the matlab directory along with associated shell scripts used to run the mappers; they will not be described here. It is recommended that the python script interface be used to run your mapper and DDiMAP even if a single iteration is desired.

The DDiMapIterate.py script may be completely configured using a configuration file placed in the current working directory in which the script is invoked or in a location specified by the -c command line parameter. Overrides may be provided in a command line format for ease of scripting multiple runs. The output of "python DDiMapIterate.py --help" is complete and somewhat daunting at first, but the bulk of these parameters are typically set in the config file.

Typical Config File

```
[Files]
outputDir = output           ; directory to contain output, relative path ok
mappingRefSeqFiles = ./myRefs.fa ; comma separated list of fasta file(s) containing
                             reference sequences for mapping
junctionRefSeqFiles =        ; optional, used only if sample prep included amplicon
                             concatenation, fasta file(s) of amplicon reference sequences
pairedEnd = False            ; set to True for use with paired end reads
fastqFiles = ./myReads.fq    ; single file or comma separated list of two files (if
                             pairedEnd is true and if mapper uses two files)

[Settings]
alignerOrder = C             ; valid chars are B (BFAST), C (CUSHAW3), S (SHRiMP2), W
                             (BWA-MEM), N (Novoalign)
firstIter = 1                ; can restart using this
maxIters = 10                ; MAXIMUM number of iters (will be adjusted to accomodate
                               length of alignerOrder)
readLength = 50              ; nominal max read length (used to make junction sequences)
readType = NT                ; read type is 'CS' (ABI SOLiD) or 'NT' (Illumina, etc.)  Few
                               aligners can handle CS.
nProcs = 8                   ; number of processors to use
reqFragConv = False          ; Set to True if you want the Frags to converge as well as
the SNVs

[DDiMap]
minAbsoluteCover=2           ; threshold used for assembling frags
fragMakerThresh=0.01         ; word frequency threshold used for accepting words for
                             making frags
fragThresh=0.1               ; threshold used for assembling frags using unverified cores
roaSize = 32                 ; must be even, if odd it will be incremented
SNVthresh=0.000400           ; threshold used for keeping words for SNV candidate
                             identification
SNVtype2thresh=0.001600      ; threshold used for type 2 SNV candidate identification
SNVtype3thresh=1.0           ; threshold used for type 3 SNV candidate identification
useDI = False                ; reads with CIGARs containing both I and D are processed

[MapperOptions]
cushawOpts =                 ; extra options for cushaw3
shrimpOpts =                 ; extra options for shrimp2 gmapper step, for illumina data
                             use --qv-offset 33
bwaMemOpts =                ; extra options for bwa-mem
novoOpts =                  ; extra options for novoalign
```

In this file, info beyond a semicolon on each line is a comment. The names of the parameters may not be changed, but the value to the right of the equal sign may be edited to suit your needs. If you wish to use a default value of a parameter, a “#” in the first column will comment out the entire line. Certain parameters are required - the output directory (outputDir), the mapping reference sequence files (mappingRefSeqFiles), the read data fastq files (fastqFiles) - and so if they are not provided in the config file, they must be provided on the command line.

How to Run DDiMapIterate.py

Set up your environment

Make sure you can access the code you need. You or your sysadmin may need to create shortcuts to files and/or modify your environment variables.

To find out if you can access the code, at a command line, run the following command, adding your mapper command(s) at the end of the list (I left in the SHRiMP2 "gmapper" command as an example)

```
$ which DDiMAP samtools bamtools gmapper
```

All the programs it is able to find are in your PATH and will appear in the command output. If one is missing, you need to fix it.

For example, if you installed DDiMAP in your home directory,

```
$ export PATH=$PATH:$HOME/DDiMAP/bin
```

will modify your PATH to include the directory containing DDiMAP for the terminal session you are running.

Now try to run DDiMAP to see if all the libraries it needs are in place. It is not unusual to have some missing runtime libraries depending on how your system is configured.

```
$ DDiMAP --help
```

If the libraries are all available you will see the help output. If not, it will complain about not finding a particular .so file, and you will need to give it a hand. One way that works is to use the LD_LIBRARY_PATH environment variable. For example,

```
$ export LD_LIBRARY_PATH=$LD_LIBRARY_PATH:/usr/local/lib/bamtools
```

will modify the search path for the current session to enable access to bamtools.

These and other examples are provided in the DDiMAPpathSetup.sh shell script in the scripts directory. You may edit this script to correspond to the locations of code on your system and then run it using the command (your path will vary at the command line and in the script).

```
$ source $HOME/DDiMAP/scripts/DDiMAPpathSetup.sh
```

Set up your data directory structure

The examples provided in the data directory provide a typical pattern. You will have a directory (or a subdirectory of a directory) with a set of read data in fastq file(s), reference sequence data in fasta file(s), and one or more DDiMapIterate config files that you have edited to set up one or more runs. An output directory associated with each of

these config files will be created, typically as subdirectories of the read data directory, but they may be placed anywhere you choose by specifying an appropriate path. This output directory may be defined in the config file or by using a -o /path/to/your/outputDir command line option.

Run the DDiMapIterate.py script

At the command line, you will invoke python and direct it to run the script. Python needs to be told where the script is located using either an absolute or relative path - your path may vary, mine is $HOME/DDiMAP/bin/ DDiMapIterate.py; when running the examples in the data directory such as "data/IterativeTest", a relative path of ../../bin/DDiMapIterate.py can be used.

```
$ python /path/to/DDiMapIterate.py
```

If no additional command line arguments are provided, the DDiMAP.cfg file in the present working directory is used if present. If the file is named differently, specify it using the -c option.

```
$ python /path/to/DDiMapIterate.py –c /path/to/myConfig.cfg
```

How to interpret the output files

Coverage File (coverage.csv)

This file, when opened in a spreadsheet program such as Excel or Libre Office Calc shows three columns of data. The first contains the reference sequence ID, the second the one-based location within that reference sequence, and the third contains the average coverage obtained after DDiMAP filtering from the two ROA collection start position analyses. For example, the first few lines look like

RefSeqID	Loc	Coverage
VH1-69-01-J1	9	2203
VH1-69-01-J1	10	2203
VH1-69-01-J1	11	2203
VH1-69-01-J1	12	2203
VH1-69-01-J1	13	2203
VH1-69-01-J1	14	2203
VH1-69-01-J1	15	2203
VH1-69-01-J1	16	2203
VH1-69-01-J1	17	6517

Variant File (snv.csv)

This file, when opened in a spreadsheet program, shows 8 columns of data.

1. reference sequence ID
2. call reason (1 = verified at both starts, 2=verified at one start, 3=not verified but present at high frequency),
3. location of the variant (relative to provided reference sequence),
4. reference base at that location,
5. variant base call at that location,
6. frequency at which the variant is present,
7. section of the reference sequence centered at the upper case variant location,
8. coverage at that location (as the average from the 2 start sites).

1	2	3	4	5	6	7	8
RefSeqID	CallReason	Loc	RefBase	CallBase	Freq	LocalSeq	Coverage
Bcl2	1	41	T	C	0.345277	`tcctgcggattgacatCtctgtgaagcagaagt`	153.5
Bcl2	1	45	G	T	0.345277	`gcggattgacatttctTtgaagcagaagtctgg`	153.5
Bcl2	1	61	G	A	0.380567	`gtgaagcagaagtctgAgaatcgatctggaaat`	247
Bcl2	1	104	G	C	1	`tttactccctctccccCcgactcctgattcatt`	239.5
Bcl2	2	113	G	C	0.0952381	`tctccccgcgactcctCattcattgggaagttt`	252
Bcl2	2	164	A	G	0.351852	`agagtgctgaagattgGtgggatcgttgcctta`	324
Bcl2	1	179	T	G	0.307851	`gatgggatcgttgcctGatgcatttgttttggt`	242

Dictionary File (dictionary.csv)

The dictionary file contains multiple columns of information for each of the words that pass the frequency threshold(s) that were set in the config file, regardless of their verification status. These columns contain:

1. Reference sequence ID
2. 1st base identifying the start position of the ROA
3. Word
4. Total coverage of the ROA
5. Edit distance of the word from reference sequence (# of base changes from ref)
6. Is the left ‘half’ of the word verified above threshold for fragment generation?
7. Is the right ‘half’ of the word verified above threshold for fragment generation?
8. Is the left ‘half’ of the word verified above threshold for SNV call?
9. Is the right ‘half’ of the word verified above threshold for SNV call?
10. # occurrences of the word (total word coverage)
11. Word coverage – forward sequence direction
12. Word coverage – reverse sequence direction
13. Count of reads with no indels matching the word
14. Count of reads with deletions but no insertions matching the word
15. Count of reads with insertions but no deletions matching the word
16. Count of reads with both insertions and deletions matching the word

The example below was created using a DDiMAP dictionary verbosity setting of 1. A setting of 0 removes the four CIGAR count data columns. A setting of 2 adds columns showing coverage contributions of the various fragments for that reference sequence to the word coverage. Note that the presence of indels in the CIGAR string type does not

mean that the indels were in the portion of the read used to generate the word, only that the read contained an indel somewhere in its sequence.

1	2	3	4	5	6	7	8	9	10	11	12	13	14	15	16
RefSe	ROAs	Sequence	ROAc	EditI	LVe	RVe	LVe	RVe	Worc	FwdC	RevC	#NoI	#DelC	#InsC	#InsA
Bcl2	9	GATCTCCGGTTGGGATTCCTGCGGATTGACAT	378	0	0	0	0	1	378	348	30	363	14	1	0
Bcl2	17	GTTGGGATTCCTGCGGATTGACATcTCTtTGA	119	2	0	0	0	1	44	28	16	42	2	0	0
Bcl2	17	GTTGGGATTCCTGCGGATTGACATTTCTGTGA	119	0	0	0	0	1	71	44	27	69	1	1	0
Bcl2	17	GTTGGGATTCCTGCGGATTGACATTTCTtTGA	119	1	0	0	0	0	4	2	2	4	0	0	0
Bcl2	25	TCCTGCGGATTGACATcTCTtTGAAGCAGAAG	106	2	1	1	1	1	39	19	20	39	0	0	0
Bcl2	25	TCCTGCGGATTGACATTTCTGTGAAGCAGAAG	106	0	1	1	1	1	67	31	36	67	0	0	0
Bcl2	33	ATTGACATcTCTtTGAAGCAGAAGTCTGaGAA	102	3	1	1	1	1	31	7	24	31	0	0	0
Bcl2	33	ATTGACATTTCTGTGAAGCAGAAGTCTGGGAA	102	0	1	1	1	1	71	23	48	69	2	0	0
Bcl2	41	cTCTtTGAAGCAGAAGTCTGaGAATCGATCTG	99	3	1	1	1	1	36	14	22	36	0	0	0
Bcl2	41	TTCTGTGAAGCAGAAGTCTGGGAATCGATCTG	99	0	1	1	1	1	63	17	46	61	2	0	0
Bcl2	49	AGCAGAAGTCTGaGAATCGATCTGGAAATCCT	139	1	1	1	1	1	60	25	35	58	2	0	0
Bcl2	49	AGCAGAAGTCTGGGAATCGATCTGGAAATCCT	139	0	1	1	1	1	79	23	56	78	1	0	0

Words that are not verified at the fragment inclusion threshold are not used for fragment generation unless they exceed the associated unverified inclusion threshold, typically set below 1.0 so that words that appear at high frequency may be used in mapping to build coverage in highly mutated regions.

SNV candidates are typically identifed using words that are fully verified using dictionaries formed at the variant calling threshold. Words that are not verified at the variant calling threshold may only be used for variant calling if they exceed the associated unverified word threshold. We recommend that this threshold be set at 1.0 so that no such identifications are made in the snv.csv file with call reason 3.

Words that occur within a single ROA that are verified at the variant calling threshold are useful for examining the number and relationship of local allelic variants. In the sequence field, the variant(s) are shown in lowercase.

Fragment File (fasta.fa)

DDiMAP generates these sequence fragments for the purpose of augmenting the reference sequences for iterative remapping. Each fragment is associated with a reference sequence and its position therein. These values, along with a counter, are combined to form a unique identifier for each fragment using a pattern of <refSeqID>_Frag_<startPosition>_<fragCounter> to enable DDiMAP post-remapping analysis. For example, the first few lines of such a file look like this:

```
>Bcl2_Frag_1_1
GCTCTTGAGATCTCCGGTTGGGATTCCTGCGGATTGACATCTCTTTGAAGCAGAAGTCTGAGAA
>Bcl2_Frag_1_2
GCTCTTGAGATCTCCGGTTGGGATTCCTGCGGATTGACATTTCTGTGAAGCAGAAGTCTGGGAA
```

```
>Bcl2_Frag_9_3
GATCTCCGGTTGGGATTCCTGCGGATTGACATCTCTTTGAAGCAGAAGTCTGAGAATCGATCTG
>Bcl2_Frag_9_4
GATCTCCGGTTGGGATTCCTGCGGATTGACATTTCTGTGAAGCAGAAGTCTGGGAATCGATCTG
>Bcl2_Frag_17_5
GTTGGGATTCCTGCGGATTGACATCTCTTTGAAGCAGAAGTCTGAGAATCGATCTGGAAATCCT
>Bcl2_Frag_17_6
GTTGGGATTCCTGCGGATTGACATTTCTGTGAAGCAGAAGTCTGGGAATCGATCTGGAAATCCT
>Bcl2_Frag_25_7
TCCTGCGGATTGACATCTCTTTGAAGCAGAAGTCTGAGAATCGATCTGGAAATCCTCCTAATTT
>Bcl2_Frag_25_8
TCCTGCGGATTGACATTTCTGTGAAGCAGAAGTCTGGGAATCGATCTGGAAATCCTCCTAATTT
```

Enhanced Reference Sequence File (refSeqEnhanced.fa)

The enhanced reference sequence file includes the original reference sequences, using an ID line pattern of <refSeqID>_Ref, the generated fragments from the fasta.fa file of the previous iteration, and optionally, chimeric junction sequences that may be present in the specimen library using an ID line pattern of Junction_<tailRefSeqID>_<headRefSeqID>. These are present only if junctionRefSeqFiles are provided. They may be useful if amplicons are concatenated prior to shearing in DNA library preparation as they represent in-vitro tail to head recombinants which would lead to mapping and interpretation difficulties, especially when full head-to-tail ("global") mapping of reads is used rather than soft clipping ("semi-local"). Reads mapped to these junctions are not currently used in DDiMAP analysis as they typically are dominated by primers used to generate the amplicons.
Sample lines from such a file would look like this:

```
>Bcl2_Ref
GCTCTTGAGATCTCCGGTTGGGATTCCTGCGGATTGACATTTCTGTGAAGCAGAAGTCTG
GGAATCGATCTGGAAATCCTCCTAATTTTTACTCCCTCTCCCCGCGACTCCTGATTCATT
...
TCGCCGCTGCAGACCCCGGCTGCCCCCGGCGCCGCCGCGGGGCCTGCGCTCAGCCCGGTG
CCACCTGTGGTCCACCTGACCCTCCGCCAGGCCGGCGACGACTTCTCCCGCCGCTACC
>Bcl6_Ref
...
>Bcl2_Frag_25_7
TCCTGCGGATTGACATCTCTTTGAAGCAGAAGTCTGAGAATCGATCTGGAAATCCTCCTAATTT
...
>Junction_Bcl6_Bcl2
TCGTGTCTACTATTTCCTTTCAGAGCCGTGATCTTCCTAATGAGAGCCGGCTCTTGAGAT
CTCCGGTTGGGATTCCTGCGGATTGACATTTCTGTGAA
```

Advanced Topic: How to Add a New Mapper to the Python Script

If you are not using one of the mapper configurations provided in the distributed DDiMAPIterate.py script, you will need to edit the script to provide information needed to run the mapper and add information regarding any parameters provided from the

configuration file and/or command line. The areas in which changes need to be made are described below. Needless to say, saving the modified script under a new name or at least keeping a backup copy is highly recommended...

Mapper abbreviation:

A single character abbreviation needs to be provided in this line of code following the format provided.

```
mapperAbbrs = {'C':'cushaw', 'S':'shrimp', 'B':'bfast', 'W':'bwa-mem', 'N':'novoalign'}
```

Commands needed to run the mapper:

A set of command line prototypes must be provided for each scenario. A scenario includes the mapper, read type NT or CS (nucleotide space or color space) indicating the form of the data (all but SOL1D NGS platforms use NT), and a single or paired end data indicator (P or S). These go into the aligner_dict name/value paired list following the pattern in the examples shown below. In these lines, there are variables that are substituted when the command is to be run (in the examples below, these are DDiFasta, DDiFastq1, DDiFastq2, DDiProcs, DDiSAM for example, more on this later). Multiple command lines are needed for some mappers, typically one or more for index generation and one or more for alignment using this index. The complete set of specifications for the currently supported configurations are included.

These command lines must account for data compatibility (CS vs NT and P vs S) as well as DDiMAP compatibility, including output to a SAM formatted file and output of only one example of alignment per read in that file if by chance a read aligns equally well to more than 1 separate locations in the reference sequences provided. In cases of a tie, it is preferable that the mapper be directed to randomly break the tie if it does not do so by default.

```
aligner_dict = {
        'C,CS,S':[
            'cushaw3 index DDiFasta -c -p bwtindex',
            'cushaw3 calign -r bwtindex -f DDiFastq1 -t DDiProcs -multi 1 CushawOpts -o DDiSAM'
            ],
        'C,NT,S':[
            'cushaw3 index DDiFasta -p bwtindex',
            'cushaw3 align -r bwtindex -f DDiFastq1 -t DDiProcs -multi 1 CushawOpts -o DDiSAM'
            ],
        'C,NT,P':[
            'cushaw3 index DDiFasta -p bwtindex',
            'cushaw3 align -r bwtindex -q DDiFastq1 DDiFastq2 -t DDiProcs -multi 1 CushawOpts -o DDiSAM'
            ],
        'S,CS,S':[
            'gmapper-cs -N DDiProcs -Q -o 1 --strata --all-contigs ShrimpOpts DDiFastq1 DDiFasta > DDiSAM'
            ],
        'S,NT,S':[
            'gmapper-ls -N DDiProcs -Q -o 1 --strata --all-contigs ShrimpOpts DDiFastq1 DDiFasta > DDiSAM'
            ],
        'S,NT,P':[
            'gmapper-ls -N DDiProcs -Q -o 1 --strata --all-contigs ShrimpOpts -1 DDiFastq1 -2 DDiFastq2 DDiFasta >
DDiSAM'
            ],
          'W,NT,S':[
```

```
            'bwa index DDiFasta',
              'bwa mem -t DDiProcs BwaMemOpts DDiFasta DDiFastq1 > DDiSAM'
            ],
          'W,NT,P':[
            'bwa index DDiFasta',
              'bwa mem -t DDiProcs BwaMemOpts DDiFasta DDiFastq1 DDiFastq2 > DDiSAM'
            ],
          'N,NT,S':[
              'novoindex DDiNIX DDiFasta',
            'novoalign -r Random -n 100 -o SAM -d DDiNIX -f DDiFastq1 > DDiSAM'
            ],
          'N,NT,P':[
              'novoindex DDiNIX DDiFasta',
            'novoalign -r Random -n 100 -o SAM NovoOpts -d DDiNIX -f DDiFastq1 DDiFastq2 > DDiSAM'
            ],
          'B,CS,S':[
              'bfast fasta2brg -f DDiFasta -A 0',
              'bfast fasta2brg -f DDiFasta -A 1',
              'bfast index -f DDiFasta -m 1111111111111111111111 -w 14 -i 1 -A 1 -n DDiProcs',
              'bfast index -f DDiFasta -m 111110100111110011111111111 -w 14 -i 2 -A 1 -n DDiProcs',
              'bfast index -f DDiFasta -m 10111111011001100011111000111111 -w 14 -i 3 -A 1 -n DDiProcs',
              'bfast index -f DDiFasta -m 1111111100101111000001100011111011 -w 14 -i 4 -A 1 -n DDiProcs',
              'bfast index -f DDiFasta -m 111111110001111110011111111 -w 14 -i 5 -A 1 -n DDiProcs',
              'bfast index -f DDiFasta -m 11111011010011000011000110011111111 -w 14 -i 6 -A 1 -n DDiProcs',
              'bfast index -f DDiFasta -m 1111111111110011101111111 -w 14 -i 7 -A 1 -n DDiProcs',
              'bfast index -f DDiFasta -m 111011000011111111001111011111 -w 14 -i 8 -A 1 -n DDiProcs',
              'bfast index -f DDiFasta -m 1110110001011010011100101111101111 -w 14 -i 9 -A 1 -n DDiProcs',
              'bfast index -f DDiFasta -m 111111001000110001011100110001100011111 -w 14 -i 10 -A 1 -n DDiProcs',
              'bfast match -f DDiFasta -A 1 -i 1-10 -k 18 -K 100000 -w 0 -t -n DDiProcs -Q 100000 -l -r DDiFastq1 >
DDiBMF',
              'bfast localalign -f DDiFasta -m DDiBMF -A 1 -n DDiProcs -U -q 20 -Q 100000 -t > DDiBAF',
              'rm DDiBMF',
              'bfast postprocess -f DDiFasta -i DDiBAF -o DDiAligned -O 1 -a 3 -z -n DDiProcs -q 20 -Q 100000 -t >
DDiSAM',
              'rm DDiBAF'
              ]
        }
```

Mapper specific optional parameters:

A novel command line option may be added to provide access to this feature from the command line by adding a new line in the parser definition section following the pattern shown for the currently supported mappers. This is not a required feature.

```
    parser.add_argument('--cushaw_opts', type=str, metavar="'options'", help='cushaw
specific options', dest='cushawOpts')
    parser.add_argument('--shrimp_opts', type=str, metavar="'options'", help='shrimp
specific options', dest='shrimpOpts')
    parser.add_argument('--bwamem_opts', type=str, metavar="'options'", help='bwa-mem
specific options', dest='bwaMemOpts')
    parser.add_argument('--novo_opts', type=str, metavar="'options'", help='novoalign
specific options', dest='novoOpts')
```

Variable substitutions:

A name/value paired list is defined in which a new entries can be added as needed. Values only used when more than one fastq file is provided are set in the "if" clause below the initial definition. These variable substitutions are made in the aforementioned command line prototypes at the time the script is run.

```
    # set substitutions for aligner commands
    commandsubs={'DDiFastq1':fastqFiles[0],
                 'DDiProcs':nProcs,
                 'DDiFasta':enhancedFastaFile,
                 'DDiBMF':thisAligned + '.bmf',
```

```
                'DDiBAF':thisAligned + '.baf',
                'DDiSAM':thisAligned + '.sam',
                'DDiNIX':thisAligned + '.nix',
                'DDiAligned':thisAligned,
                'CushawOpts':cushawOpts,
                'ShrimpOpts':shrimpOpts,
                'BwaMemOpts':bwaMemOpts,
                'NovoOpts':novoOpts}

if (len(fastqFiles) > 1):
    commandsubs['DDiFastq2']=fastqFiles[1]
```
