# Supplementary material for: Ultradeep analysis of tumor heterogeneity in regions of somatic hypermutation
Source: Genome Med. 2015 Mar 12;7(1):24. doi: 10.1186/s13073-015-0147-1 (PMC4395979; doi:10.1186/s13073-015-0147-1)
Supplement: Additional file 4: — Supplemental Data: contains supplemental data tables and figures. [file 13073_2015_147_MOESM4_ESM.pdf]

Table AF1. SNV identified by DDiMAP according to sample type and gene target, with *BCL2* and *BCL6* showing differential mutation rates between FL specimens and non-malignant lymph node controls.

Targets	Follicular Lymphoma Specimens												Controls: Reactive LN			Controls: Cell line	Total SNVs
	126	127	128	129	131	132	133	134	135	136	137	139	140	155	158	293	
**SNV not IGH**	41	18	190	57	30	81	15	90	15	54	29	39	6	9	4	9	686
**at AID motif***	21	6	107	27	15	38	4	50	9	29	12	19	2	1	1	1	342
**Total SNV**	310	102	242	192	153	306	181	527	112	266	219	50	NA	NA	NA	NA	2670
BCL2	23	2	101	35	17	36	5	54	4	37	9	17	**0**	**0**	**0**	**0**	340
**% freqMFC@**	10%	14%	24%	20%	25%	20%	20%	11%	10%	45%	30%	30%					
≥MFC	15	2	54	33	10	29	5	13	4	11	8	17					201
≤3%	4	0	30	0	7	7	0	41	0	23	1	0					113
≤1%	1	0	24	0	6	3	0	24	0	20	1	0					79
BCL6	5	2	39	3	5	7	2	13	3	3	5	6	**0**	**0**	**0**	4	99
RHOH	4	2	16	2	2	4	1	3	1	2	1	1	1	**0**	**0**	1	42
PAX5	2	1	6	**0**	2	7	1	5	1	4	1	2	**0**	**0**	**0**	1	33
***IGH*-enh**	**0**	3	9	14	**0**	17	1	12	4	4	11	8	1	1	2	**0**	85
CD83	1	1	7	**0**	**0**	2	2	**0**	1	**0**	1	1	1	**0**	**0**	**0**	16
***MYC* TSS-1**	4	2	**0**	1	1	2	**0**	1	**0**	3	1	2	1	2	2	2	23
***MYC* TSS-2**	2	4	1	1	2	2	1	1	**0**	**0**	**0**	**0**	**0**	2	**0**	**0**	15
PIM1	**0**	1	11	**0**	**0**	4	2	1	**0**	**0**	**0**	2	**0**	2	**0**	1	23
SYK	1	**0**	**0**	1	1	**0**	**0**	**0**	1	1	**0**	**0**	2	2	**0**	**0**	9
***IGH*€**	269	84	52	135	123	225	166	437	97	212	190	11	NA	NA	NA	NA	2001
**Total bases**	11756	12233	11853	11752	11389	11952	11449	11927	11789	12630	11719	11727	10129	10129	10129	10129	

* excludes IGH results

@ frequency of most frequent clone (MFC) based on minimum estimate from *BCL2*

€ IGH SNV data reflects differences from the specimen-specific Sanger-level IGH sequence, not from germline reference sequence.

Gene regions as described in methods.

TSS – Transcription Start Site

A	Non-mutated C + G	SNV at C + G	Totals
C + G AID motif	537	51	588
C + G Non-motif	3450	78	3528
Total C + G	3987	129	4116

$p < 0.0001$

D	Non-mutated A + T	SNV at A + T	Totals
A + T WA/TW	2944	152	3096
A + T Non-motif	2343	57	2400
Total A + T	5287	209	5496

$p < 0.0001$

B	Non-mutated C + G	SNV ≥15% C + G	Totals
C + G AID motif	560	28	588
C + G Non-motif	3487	41	3528
Total C + G	3987	69	4116

$p < 0.0001$

E	Non-mutated A + T	SNV ≥15% A + T	Totals
A + T WA/TW	3016	80	3096
A + T Non-motif	2366	34	2400
Total A + T	5382	114	5496

$p < 0.0015$

C	Non-mutated C + G	SNV ≤1% C + G	Totals
C + G AID motif	577	11	588
C + G Non-motif	3513	15	3528
Total C + G	4090	26	4116

$p < 0.0001$

F	Non-mutated A + T	SNV ≤1% A + T	Totals
A + T WA/TW	3045	51	3096
A + T Non-motif	2322	78	2400
Total A + T	5367	129	5496

$p < 0.0011$

Figure AF1. ***BCL2*** **SNV at high and low frequency are consistent with somatic hypermutation patterns.** Tables A-C detail SNVs at C and G for their consistency with the canonical AID motif, defined as C/G at the underlined base in WRCY/RGYW sites changed to either A/T or G/C. Tables D-F detail SNVs patterns at A and T for their consistency with the *POLH* mutation patterns, defined as A>X or T>X at underlined base in WA/TW sites. Tables A and D show total SNV data, while B and E use only at SNVs at frequencies ≥15%, while C and F use only SNV frequencies at ≤1%. All SNV data show a highly significant and consistent tendency to the AID-mediated mutation patterns, implying that the extremely low frequency SNVs calls represent a biological process and are not due to analytical error. The Fisher's exact one-tailed analysis performed at http://graphpad.com.

Table AF2. Evolution of *IGHV* FR1-FR3 sequence from SOLiD data.

	Method	BFAST			SHRiMP2			BSBSn converged			CUSHAW3			CUSHAW3 converged		
Specimen	SNV*/Total bases	TP	FN	FP	TP	FN	FP	TP	FN	FP	TP	FN	FP	TP	FN	FP
128	18/274 (10.3%)	13	5	0	15	3	0	18	0	0	18	0	0	18	0	0
134	47/280 (16.8%)	16	31	3	21	26	1	21	26	3	30	17	5	38	9	5
136	48/286 (16.8%)	12	36	1	16	32	1	15	33	1	37	11	0	42	6	1
132	50/280 (17.9%)	20	30	1	15	35	0	31	19	6	16	34	0	40	10	0
	Method PPV	.92			.97			.89			.95			.96		
	Method FNR	.63			.59			.48			.38			.15		

*Single Nucleotide Variation (SNV) from germline determined by Sanger sequencing
TP = total positive base calls
FN = false negative base calls
FP = false positive base calls
Positive predictive rate (PPV) = TP/(TP+FP)
False negative rate (FNR) = FN/SNV
